# Psychiatric genetic liability is associated with the severity of COVID-19 and other acute respiratory infections: an observational study across five Northern European countries

**DOI:** 10.1186/s13073-026-01723-w

**Published:** 2026-07-28

**Authors:** Kadri Kõiv, Ragna Bugge Askeland, Lea Arregui Nordahl Christoffersen, Ingibjörg Magnúsdóttir, Daniel McCartney, Ole A. Andreassen, Thor Aspelund, Sofiya Babok, Mie Topholm Bruun, Archie Campbell, Elizabeth C. Corfield, DBDS Genetic Consortium, Maria Didriksen, Christian Erikstrup, Estonian Biobank Research Team, Andres Metspalu, Lili Milani, Tõnu Esko, Mari Nelis, Georgi Hudjashov, Arna Hauksdóttir, Jóhanna Jakobsdóttir, Bitten Aagaard Jensen, Isabella Loft, Reedik Mägi, Dorte Helenius Mikkelsen, Sisse Rye Ostrowski, Johanne Hagen Pettersen, Liam Quinn, Erik Sørensen, Edda Bjork Thordardottir, Yue Wang, Thomas Werge, Helga Ask, Ole Birger Vesterager Pedersen, Unnur Anna Valdimarsdóttir, Kelli Lehto

**Affiliations:** 1 Estonian Genome Centre, Institute of Genomics, University of Tartu, Tartu, Estonia; 2 PsychGen Centre for Genetic Epidemiology and Mental Health, Norwegian Institute of Public Health, Oslo, Norway; 3 NORMENT Centre, Institute of Clinical Medicine, University of Oslo, Oslo, Norway; 4 Institute of Biological Psychiatry, Mental Health Services, Amager and Hvidovre Hospital, Copenhagen University Hospital, Roskilde, Denmark; 5 Department of Clinical Immunology, Zealand University Hospital, Koege, Denmark; 6 Centre of Public Health Sciences, Faculty of Medicine, School of Health Sciences, University of Iceland, Reykjavik, Iceland; 7 Centre for Genomic and Experimental Medicine, Institute of Genetics and Cancer, University of Edinburgh, Western General Hospital, Edinburgh, UK; 8 Division of Mental Health and Addiction, Oslo University Hospital, Oslo, Norway; 9 Department of Clinical Immunology, Odense University Hospital, Odense, Denmark; 10 Psychiatric Genetic Epidemiology Group, Research Department, Lovisenberg Diaconal Hospital, Oslo, Norway; 11 MRC Integrative Epidemiology Unit, Population Health Sciences, Bristol Medical School, University of Bristol, Bristol, UK; 12 Department of Clinical Immunology, Copenhagen University Hospital, Rigshospitalet, Copenhagen, Denmark; 13 Department of Clinical Immunology, Aarhus University Hospital, Aarhus, Denmark; 14 Department of Clinical Medicine, Aarhus University, Aarhus, Denmark; 15 Department of Clinical Immunology, Aalborg University Hospital, Aalborg, Denmark; 16 Department of Clinical Medicine, Faculty of Health and Medical Sciences, University of Copenhagen, Copenhagen, Denmark; 17 PROMENTA Research Center, Department of Psychology, University of Oslo, Oslo, Norway; 18 Unit of Integrative Epidemiology, Institute of Environmental Medicine, Karolinska Institutet, Stockholm, Sweden; 19 Department of Epidemiology, Harvard T H Chan School of Public Health, Boston, MA USA

**Keywords:** Genetic predisposition, Polygenic scores, Mental health, Acute respiratory infection, Psychiatric disorders, Acute respiratory infection symptoms, COVID-19, Community samples

## Abstract

**Background:**

Pre-existing psychiatric disorders have been associated with the severity of acute respiratory infections, including COVID-19, particularly in hospitalized populations. However, the underlying mechanisms, especially in community-based populations, remain unknown, limiting preparedness for future pandemics. We investigated the role of genetic liability for psychiatric disorders and related traits in COVID-19 and other respiratory infection severity among individuals reporting SARS-CoV-2 testing and available respiratory symptom data.

**Methods:**

We included population-based cohort data from Denmark, Estonia, Iceland, Norway, and the United Kingdom (N = 78,507; 62% female; 41% SARS-CoV-2-positive; May 2020—March 2023). Respiratory infection severity was defined by self-reported days bedridden and an eleven-symptom composite score during the acute illness phase. Polygenic scores (PGSs) for eight psychiatric disorders and the personality trait neuroticism indexed genetic liability. Meta-analysed estimates from stepwise-adjusted regression analyses controlling for education, lifestyle, and psychiatric and somatic diagnosis history were used to assess associations between PGSs and acute respiratory infection severity, stratified by COVID-19 status.

**Results:**

Higher genetic liability to insomnia, major depression (MD), and neuroticism was consistently associated with longer time bedridden (relative risk ratios per 1 SD change in PGS: 1.07–1.14, 95% confidence intervals [CIs]: 1.03–1.21) and more symptoms (incident rate ratios [IRRs]: 1.02–1.04, 95% CIs: 1.01–1.05) due to COVID-19. PGSs for insomnia, MD, neuroticism, anxiety disorder, bipolar disorder, and schizophrenia were also linked to higher non-COVID-19 symptom count (IRRs: 1.01–1.05, 95% CIs: 1.00–1.07). These associations remained robust after adjustment for lifestyle, education and medical comorbidities. Individuals in the highest MD PGS decile reported 20% more symptoms in non-COVID-19 illness and 10% more in COVID-19 compared with the bottom decile. No consistent associations were detected with attention-deficit/hyperactivity disorder, alcohol dependence and post-traumatic stress disorder. No genetic link was observed with ischaemic heart disease or type 2 diabetes.

**Conclusions:**

Shared genetics may contribute to the link between psychiatric conditions and respiratory infection severity in community settings. Our results underscore the role of psychiatric genetic liability, beyond diagnosed psychiatric disorders, in contributing to both COVID-19 and other respiratory infection severity. These findings provide insights that may improve future risk stratification and public health strategies targeting respiratory viruses.

**Supplementary Information:**

The online version contains supplementary material available at https://doi.org/10.1186/s13073-026-01723-w.

## Background

The coronavirus disease of 2019 (COVID-19) pandemic brought an unprecedented focus to the bidirectional links between acute respiratory infections (ARIs) and mental health. To date, substantial evidence supports the association between pre-existing psychiatric disorders and increased severity of ARIs [1, 2] with severe acute respiratory syndrome coronavirus 2 (SARS-CoV-2) being no exception. Several meta-analyses have found that individuals with a history of psychiatric disorders, including mood disorders, substance use disorders, and schizophrenia, are at increased risk of a more severe course of COVID-19, including hospitalization and mortality [3–5].

Despite the accumulating evidence on the role of mental health morbidities in risks of hospitalization and death due to COVID-19 and other ARIs, the evidence in non-hospitalized population samples, which constitutes the majority of affected individuals, remains scarce. The clinical presentation of ARIs varies significantly in community settings, ranging from asymptomatic cases to more severe forms with long-term health consequences such as long-COVID with extensive recovery periods [6]. The identification of vulnerable groups could inform future preventive strategies, including prioritization in vaccination programs [7].

The COVIDMENT consortium [8], comprising approximately 393,000 participants, is a large multinational Northern European research collaboration that leverages population-based resources in Denmark, Estonia, Iceland, Norway, Sweden and the United Kingdom to investigate mental morbidity before, during, and after the COVID-19 pandemic in the general adult population as well as in specific risk groups. With harmonised questionnaire and genetic data, COVIDMENT enables large-scale analyses of the links between mental health and COVID-19 outcomes. Within this collaboration, we have previously demonstrated that greater acute COVID-19 severity is associated with higher levels and longer-lasting depressive symptoms, anxiety symptoms, and sleep disturbances in primarily non-hospitalized population samples across cohorts from six countries [9]. However, the mechanisms underlying increased risk for more severe ARI in patients with psychiatric disorders remain unclear. In addition to known risk factors, such as poor lifestyle and comorbid diseases such as cardiovascular disease and type 2 diabetes [10, 11], shared genetic liability to specific psychiatric disorders, particularly depression, schizophrenia, and bipolar disorder, has been implicated in susceptibility to and severity of COVID-19 and other acute respiratory infections, with Mendelian randomization studies suggesting partially overlapping mechanisms [12–15]. Yet, genetic studies so far have mostly focused on hospitalized populations, while reports in community samples are scarce. The individual-level polygenic scores (PGS) for common psychiatric conditions can be used to determine the role of shared genetic factors contributing to the observed link between psychiatric conditions and illness severity of acute respiratory disease. In addition, PGS-based stratification of individuals allows detection of vulnerable groups with higher risk of a more severe illness course [16, 17].

To capture genetic liability to psychiatric morbidity, we constructed PGSs for nine psychiatric disorders and related traits selected a priori based on 1) prior evidence linking them phenotypically and/or genetically to ARI susceptibility and severity, 2) covering major domains of psychiatric liability, and 3) the availability of large, well-powered GWASs to maximize PGS predictive power. These included internalizing disorders (major depression, anxiety disorder, post-traumatic stress disorder), severe mental illness with a psychosis component (bipolar disorder, schizophrenia), neurodevelopmental (attention-deficit/hyperactivity disorder), and substance use disorders (alcohol dependence). In addition, we included two transdiagnostic traits, insomnia and personality trait neuroticism, which are strongly genetically correlated with internalizing disorders [18–20] but also capture subclinical variation, stress reactivity, and processes relevant to immune function and acute illness severity [21, 22].

The aim of this study is to expand our previous results to investigate the genetic association between psychiatric disorders and related traits and ARI severity—both COVID-19 and non-COVID-19—in primarily non-hospitalized population samples from five Northern European countries. Specifically, we aim to 1) explore the role of genetic liability to common psychiatric disorders and related traits in the severity of SARS-CoV-2 infection above and beyond diagnosed psychiatric and somatic comorbidities, lifestyle factors and educational attainment, and 2) assess these associations in relation to both COVID-19 and other acute respiratory illness severity to estimate the specificity of these associations.

## Methods

### Study population and design

**Fig. 1:**
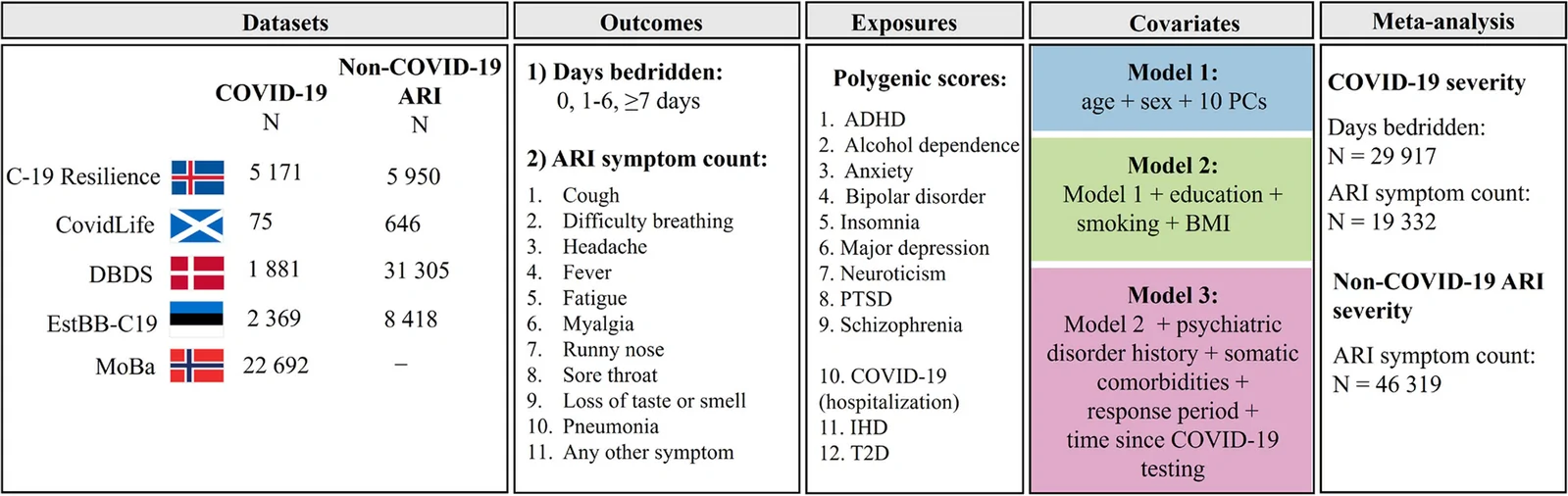
Overview of the datasets and the study design. Notes: C-19 Resilience—the Icelandic COVID-19 National Resilience Cohort; CovidLife—UK-based CovidLife study; DBDS—the Danish Blood Donor Study; EstBB-C19—the Estonian Biobank COVID-19 cohort; MoBa—the Norwegian Mother, Father, and Child Cohort study. ADHD—attention-deficit/hyperactivity disorder; ARI—acute respiratory infection; BMI—body mass index; IHD—ischaemic heart disease; PCs—genetic principal components; PTSD—post-traumatic stress disorder; T2D—type 2 diabetes mellitus

This study included SARS-CoV-2-tested individuals from the general population from five COVIDMENT consortium cohorts with harmonised data collections prospectively planned in March 2020 along with genome-wide genotyping data (Fig. 1). The cohorts were: The Icelandic COVID-19 National Resilience Cohort (C-19 Resilience) [8] with genetic data from deCODE Genetics [23], the UK-based CovidLife [24] study with genetic data from Generation Scotland [25], the Danish Blood Donor Study (DBDS) [26], the Estonian Biobank COVID-19 cohort (EstBB-C19) [27], and the Norwegian Mother, Father, and Child Cohort study (MoBa) [28]. Detailed description of the COVIDMENT cohorts can be found elsewhere [8]. All cohorts included adult participants from the general population, but used different recruitment strategies, with most recruiting from established cohorts whilst others also allowed self-recruitment via social media (C-19 Resilience, CovidLife). Ethical approvals for all included cohorts were obtained from regional or national ethics committees, and all participants provided electronic informed consent (Additional file 1: Table S1).

We excluded participants with incomplete information on ARI illness severity, COVID-19 diagnosis, key covariates or genotype data. After exclusions, 78 507 individuals were included in the final analytical sample: C-19 Resilience N = 11 121, CovidLife N = 721, DBDS N = 33 186, EstBB N = 10 787, and MoBa N = 22 692. Only complete cases for every model in the regression analyses were used.

### Procedure

The analytical sample consisted of participants with available SARS-CoV-2 testing and ARI symptom data. We used self-reports of being tested for SARS-CoV-2 together with the corresponding test result (positive or negative) and COVID-19-like symptoms, collected via questionnaires over the period of April 2020 to March 2023 (Table 1, by cohort: Additional file 1: Table S2). Participants with a positive SARS-CoV-2 q-RT-PCR test during the study period were categorized as the COVID-19 group and participants with a negative test for SARS-CoV-2 were referred to as the non-COVID-19 ARI group. This classification was applied consistently across all cohorts based on self-reported testing results. In the MoBa cohort, symptom information was available only for COVID-19 positive individuals, whereas all other cohorts recorded symptoms for both COVID-19 and non-COVID-19 ARI.

**Table 1:** Characteristics of the study population across five cohorts (total N = 78 507)

	**COVID-19**	**Non-COVID-19 ARI**	
Total sample size, N (%)	32 188 (41.0)	46 319 (59.0)	
C-19 Resilience (IS)	5 171 (16.1)	5 950 (12.8)	
CovidLife (UK)	75 (0.2)	646 (1.4)	
DBDS (DK)	1 881 (5.8)	31 305 (67.6)	
EstBB-C19 (EE)	2 369 (7.4)	8 418 (18.2)	
MoBa (NO)	22 692 (70.5)	-	
Sex			*p* < 0.001, V = 0.09
Male	10 348 (32.2)	19 119 (41.3)	
Female	21 798 (67.8)	27 200 (58.7)	
Age			*p* < 0.001, V = 0.38
* Mean age (SD)*	*47.4 (8.6)*	*50.8 (13.1)*	
18-29 years	642 (2.0)	3 492 (7.5)	
30-39 years	3 883 (12.1)	7 668 (16.6)	
40-49 years	17 656 (54.9)	9 740 (21.0)	
50-59 years	6 878 (21.4)	12 038 (26.0)	
60-69 years	2 110 (6.6)	9987 (21.6)	
70 years or older	980 (3.0)	3394 (7.3)	
Missing	19 (0.1)		
Not available for reporting^@^	20 (0.1)		
Education			*p* < 0.001, V = 0.20
Compulsory or less	1 037 (3.2)	2 153 (4.6)	
Upper secondary, vocational, or other	8 255 (25.6)	5 810 (12.5)	
Bachelor's/diploma university degree	12 140 (37.7)	25 212 (54.4)	
Master's or PhD	9 612 (29.9)	13 126 (28.3)	
Missing	1 137 (3.5)	18 (0.04)	
Not available for reporting^@^	7 (0.02)		
BMI (kg/m^2)			*p* = 0.007, V = 0.01
Normal weight or underweight (< 25)	12 488 (38.8)	19 955 (43.1)	
Overweight (25–30)	10 282 (31.9)	17 295 (37.3)	
Obese (> 30)	5 626 (17.5)	9 066 (19.6)	
Missing or not measured	3 792 (11.8)	3 (0.01)	
Current smoking			*p* < 0.001, V = 0.17
Never	23 874 (74.2)	30 899 (66.7)	
Former smoker	3124 (9.7)	10 394 (22.4)	
Current smoker	5 158 (16.0)	5 019 (10.8)	
Missing	32 (0.1)	7 (0.02)	
History of psychiatric disorders			*p* < 0.001, V = 0.07
Yes	6 563 (20.4)	12 470 (26.9)	
No	25 625 (79.6)	33 843 (73.1)	
Missing	—	7 (0.01)	
Chronic medical conditions			*p* < 0.001, V = 0.09
No condition	5 286 (16.4)	20 682 (44.7)	
1 condition	3 094 (9.6)	15 573 (33.6)	
2 conditions	978 (3.0)	6 835 (14.8)	
> 2 conditions	355 (1.1)	3 226 (7.0)	
Missing	22 475 (69.9)	3 (0.01)	
Response periods			
April-June 2020	515 (1.6)	4 763 (10.3)	
July–September 2020	190 (0.6)	1 421 (3.1)	
October-December 2020	974 (3.0)	16 356 (35.3)	
January-March 2021	1 489 (4.6)	3 976 (8.6)	
April-June 2021	2 087 (6.5)	19 151 (41.3)	
July–September 2021	#	#	
October-December 2021	#	—	
January-March 2022	—	—	
April-June 2022	—	612 (1.3)	
July–September 2022	2 611 (8.1)	40 (0.1)	
October-December 2022	1 625 (5.0)	—	
January-March 2023	#	—	
Missing or not reported	22 692 (70.5)^a^		
Illness severity – Time bedridden^b,c^			
Never bedridden	13 863 (43.1)	—	
Bedridden 1–6 days	10 102 (31.4)	—	
Bedridden 7 days or more	5 952 (18.5)	—	
Missing^a^ or not measured^c^	2 229 (6.9)	—	
Illness severity – Symptom count			*p* < 0.001, d = 0.59
*Mean symptom count (SD)*	*2.8 (2.2)*	*1.5 (2.2)*	
Missing or not measured	12 856 (39.9)^a^		

Cohen’s d and Cramer’s V were calculated excluding missing values

*SD—*standard deviation

a—Exact dates not available in the Norwegian Mother, Father, and Child Cohort study; analyses are based on data available up to October 6th, 2022

b—Days with fever in the Estonian Biobank COVID-19 cohort

c—Time bedridden not measured in the CovidLife study

d—Cohen's d

V—Cramer's V

^#^—Low case numbers (< 5), cases grouped together with the previous period

@—not available for group-wise reporting in the Danish Blood Donor Study

We used two approaches to capture acute illness severity. In COVID-19 positive individuals, we used duration of being bedridden as a proxy for illness severity, operationalised by the self-reported number of days confined to bed due to COVID-19 (classified as: not bedridden, bedridden for 1–6 days, or bedridden for 7 days or longer), as used in our previous reports [9, 29]. For EstBB-C19 only, illness severity was defined by the duration of fever (classified as: no fever, fever for 1–6 days, fever for 7 or more days). Data regarding time bedridden were available only for individuals who tested positive for COVID-19 in DBDS, C-19 Resilience and MoBa, and was not collected in CovidLife.

We used a second definition of illness severity based on a summarized score of self-reported ARI symptoms in COVID-19 patients. Eleven individual symptoms were used across cohorts, specifically: 1) cough, 2) difficulty breathing or shortness of breath, 3) headache, 4) fever, 5) fatigue, 6) muscle aches and/or joint pains, 7) runny or congested nose, 8) sore throat, 9) loss of or change in taste or smell, 10) pneumonia, and 11) any other COVID-19 related symptom reported. Each symptom was classified as present (1) or absent (0), resulting in a total symptom count ranging from 0 to 11. The available symptom composition and maximum count differed by cohort, ranging from 8 in MoBa to 11 in CovidLife and EstBB (available symptom lists by cohorts: Additional file 1: Table S3). Both symptomatic and asymptomatic individuals were retained to allow illness severity to be modelled across the full spectrum of ARI severity ranging from no symptoms to severe illness. To determine whether the associations found were specific to COVID-19, we used symptom count data from individuals with ARI symptom data but who tested negative for SARS-CoV-2.

Covariates were the following (for details, please see Additional file 1: Table S4): age (continuous, in years); sex (male, female); highest education level (compulsory or less [no formal education]; upper secondary, vocational, or other; bachelor’s or diploma university degree; and master’s or PhD); smoking (never; former smoker; current smoker); body mass index (kg/m^2, continuous); history of psychiatric disorders (self-reported in all cohorts except EstBB and DBDS where the history of psychiatric diagnoses based on electronic health records (EHR) were used; classified as yes or no); history of chronic medical conditions (defined as self-reported hypertension, diabetes, heart disease, lung disease, chronic kidney disease, cancer, immunosuppressive state or immunosuppressive therapy (classified as: no condition; one condition; two conditions; more than two conditions); response periods (in 3-month intervals from April 2020 to March 2023 [information not available in MoBa]); time since q-RT-PCR testing at the time of symptom reporting (less than 8 weeks, 2–6 months, 7 or more months [information not available in MoBa and in C-19 Resilience for non-COVID-19 ARI group]).

### Polygenic scores

Nine psychiatric disorders and related traits were selected for polygenic scoring: attention-deficit/hyperactivity disorder (ADHD), alcohol dependence (ALC_D), anxiety disorder (ANX), bipolar disorder (BIP), insomnia (INS), major depression (MD), post-traumatic stress disorder (PTSD), schizophrenia (SCZ), and "the big five" personality trait neuroticism (NEU), a known risk factor for several psychiatric conditions [18]. Additionally, three somatic PGSs were computed for validation (COVID-19 illness severity) and comparison with known risk factors for more severe ARIs (ischaemic heart disease [IHD] and type 2 diabetes mellitus [T2D]). PGSs, which are the weighted sum scores of thousands of small genetic effects based on large genome-wide association studies, were computed based on the summary results from recent and well-powered GWASs (Additional file 1: Table S5) available at the time of the beginning of the analyses (analysis period in different cohorts: 15.08.2022–18.06.2024), selected based on phenotype definition, sample size, and availability of leave-cohort-out summary statistics to avoid sample overlap between the discovery and target cohorts. The genome-wide genotyping, imputation and quality control steps in each cohort are described in Additional file 1: Supplementary Methods. PGSs were computed using the PRS-CS-auto algorithm [30] for allele weighting and PLINK 1.9 or 2.0 for allele scoring [31]. The PGSs were standardized across each cohort before statistical analyses to a scale where the mean is zero and the unit is 1 standard deviation. For decile-based analyses, individuals in each cohort were stratified into 10 equally sized groups based on PGS values, ranging from the bottom 10% to the top 10%.

### Statistical analysis

Differences between COVID-19 and non-COVID-19 ARI groups were assessed using chi-square test for categorical variables and Welch’s t-test for continuous variables, with effect sizes reported as Cramér’s V and Cohen’s d, respectively (Table 1). We estimated the relative risk ratios (RRRs) for time bedridden using multinomial logistic regression in COVID-19 patients (not bedridden vs. bedridden for 1–6 days; not bedridden vs. bedridden for 7 days or longer). We estimated the incident rate ratios (IRRs) of the symptom count using Poisson regression models separately for COVID-19 and non-COVID-19 ARI symptom count. The analytical pipeline was harmonized and run using a standardized protocol separately in each cohort. Three models were run, incrementally adjusting for additional covariates. In Model 1, we adjusted for age, sex, and the first 10 genetic principal components as technical covariates adjusting for potential population stratification. Model 2 was additionally adjusted for the following sociodemographic and lifestyle factors: education, smoking and BMI. Model 3 was additionally adjusted for history of psychiatric disorders, chronic medical conditions, response period and time since acute ARI (i.e. q-RT-PCR testing). If a covariate was not available in some cohort, the cohort ran the models with available covariates (Additional file 1: Table S4). Because of significant missingness for chronic medical conditions covariate in MoBa, a separate ‘missing’ category was used to avoid dropping a large proportion of participants from the analysis. The decile analysis was conducted only on the PGS-ARI associations that remained significant in Model 3, but to maximize sample size, Model 1 was used to estimate the IRRs for the 5th and 10th deciles as compared to the 1st. These IRRs were then pooled across cohorts using random-effects meta-analysis. The CovidLife cohort was excluded from this analysis due to its small sample size, which limited the reliability of comparisons between deciles. While the 1^st^ and the 10^th^ decile comparison allows for investigating the differences between the extreme ends of the genetic liability, the comparison between 5^th^ and 10^th^ decile investigates the differences between the population average and the highest genetic liability.

Meta-analyses were conducted by applying a random-effects model using the metafor package [32] in R (4.3.0). The *I*^2^ statistic was used to assess the heterogeneity across the cohorts and Cochran’s Q test for *p*-values. To account for multiple testing, Bonferroni correction for nine (*p* < 0.0056) or three (*p* < 0.017) tests were applied respectively for psychiatric and somatic PGS models. Data management and statistical analyses were conducted in R (version 4.3.0; R Core Team, 2023), SAS (version 9.4; SAS Institute Inc., Cary, NC, USA), and Stata BE (version 17, StataCorp LLC, College Station, TX, USA).

This study is reported according to the Strengthening the Reporting of Observational studies in Epidemiology (STROBE) checklist (Additional file 1: Table S12).

## Results

Of the 78 507 participants, 32 188 (41.0%) reported having tested positive for SARS-CoV-2 during the study period. Individuals reporting a COVID-19 infection were more likely to be female, on average younger, and have a lower prevalence of pre-existing psychiatric disorders compared to the non-COVID-19 ARI group (Table 1). In addition, the COVID-19 group reported more ARI symptoms than non-COVID-19 ARI group (mean symptom count in COVID-19 = 2.8 [SD 2.2]; non-COVID-19 ARI = 1.5 [SD 2.2]), indicating higher severity of COVID-19 illness compared to other ARIs. Among COVID-19-infected individuals, 49.9% were bedridden during acute infection (31.4% for 1–6 days; and 18.5% for 7 days or more).

The five cohorts were generally sociodemographically similar, all having over representation of females and the majority of the respondents having a university degree (Table 1, by cohort: Additional file 1: Table S2).

### Psychiatric PGSs and two definitions of COVID-19 severity

**Fig. 2:**
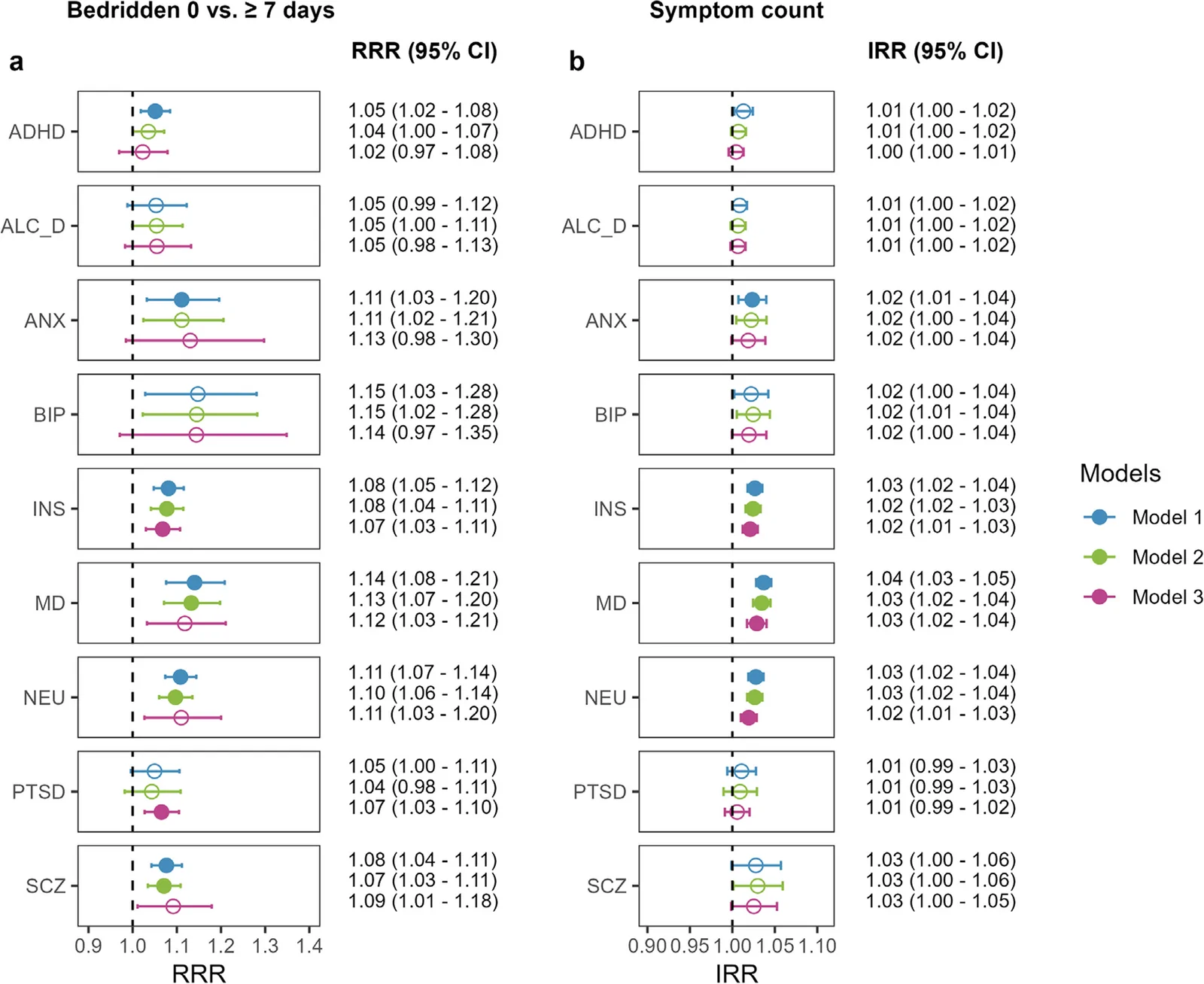
Associations between psychiatric polygenic scores and acute COVID-19 severity defined as **a** bedridden for seven or more days (vs. bedridden for 0 days, max N = 19 801 in Model 1) and **b** symptom count (max N = 19 323 in Model 1). Notes: Model 1: PGS + Age + Sex + 10 PCs; Model 2: Model 1 + Education + Smoking + BMI; Model 3: Model 2 + Previous psychiatric diagnosis + Somatic comorbidities + Response period + Time since testing. Cohorts included: **a** the Icelandic COVID-19 National Resilience Cohort, the Estonian Biobank COVID-19 cohort, the Norwegian Mother, Father, and Child Cohort study; **b** the Icelandic COVID-19 National Resilience Cohort, the UK-based CovidLife study, the Danish Blood Donor Study, the Estonian Biobank COVID-19 cohort, the Norwegian Mother, Father, and Child Cohort study. For N per phenotype, cohort and model, please refer to Additional file 1: Table S6, S7. ADHD—attention-deficit/hyperactivity disorder, ALC_D—alcohol dependence, ANX—anxiety disorder, BIP—bipolar disorder, INS—insomnia, MD—major depression, NEU—neuroticism, PTSD—post-traumatic stress disorder, SCZ—schizophrenia. Filled circles—significant after Bonferroni correction for nine psychiatric PGSs (*p* < 0.0056). ARI—acute respiratory illness, RRR—meta-analysed relative risk ratio, IRR—meta-analysed incidence rate ratio, CI—confidence intervals

The meta-analysis of associations between psychiatric PGSs and COVID-19 severity defined as time bedridden (zero vs. 7 or more days) revealed significant associations with genetic liability for most of the PGSs (ADHD, ANX, INS, MD, NEU, PTSD, and SCZ) (RRR range = 1.05–1.15 per one SD change in the PGS) (Fig. 2a; by cohort: Additional file 1: Table S6). The strongest and most consistent association across all three models were observed with the genetic liability for INS, followed by MD, NEU and SCZ which were significant in Models 1 and 2. Adjustment for education and lifestyle factors in Model 2 generally led to slight attenuation of effect sizes, and the associations for ADHD and ANX were no longer statistically significant after correction for multiple testing. In Model 3, which additionally accounted for somatic comorbidities, history of psychiatric diagnoses, and response period, we observed a greater uncertainty around the RRR estimates, and the associations for MD, NEU, and SCZ no longer reached the adjusted significance threshold. Notably, heterogeneity across cohorts was observed, particularly for BIP PGS, which showed substantial variation (*I*^2^ 76–80% across all three models, *p* < 0.05, Additional file 1: Table S9). Analyses contrasting categories zero vs. 1–6 days bedridden, indicating milder COVID-19 severity (Additional file 1: Fig. S1a), yielded similar effect directions but smaller effect sizes (RRR range = 1.05–1.08), as expected. Significant associations emerged with ANX and BIP PGSs in Models 1 and 2; however, in Model 3, only the association with BIP PGS remained statistically significant. Heterogeneity was particularly pronounced for INS PGS in these analyses (*I*2 = 71–74%, *p* < 0.05 across all three models; Additional file 1: Table S9).

The results of the meta-analyses exploring associations between COVID-19 severity defined by symptom count revealed generally similar patterns of association to the "time bedridden" definition. We detected significant associations with the genetic liability for four psychiatric PGSs, with IRRs ranging from 1.01 to 1.04 per one SD increase in the PGS (Fig. 2b for meta-analysis; Additional file 1: Table S7 by cohort). After correction for multiple testing, PGSs for ANX, INS, MD, and NEU remained significantly associated with COVID-19 symptom count in Model 1. The inclusion of education and lifestyle factors in Model 2 had minimal impact on the associations, apart from the ANX PGS, which was no longer significantly associated with COVID-19 severity. In the fully adjusted Model 3, the association estimates were generally slightly attenuated. Overall, higher genetic liability to INS, MD and NEU increased the rate of more acute symptoms for COVID-19 regardless of sociodemographic and lifestyle factors or psychiatric diagnoses and somatic comorbidities. Across cohorts, the effect directions and magnitudes were largely consistent, except for the CovidLife, which diverged from all other cohorts in some of the PGS analyses (Additional file 1: Table S7 and S8). We identified significant heterogeneity in BIP and SCZ PGS associations with COVID-19 symptom count (*I*^2^ 72–75%, 87–90%, respectively, across all three models, *p* < 0.05, Additional file 1: Table S9). However, excluding CovidLife from the meta-analysis for BIP did not improve the *I*^2^ statistic for this psychiatric PGS considerably, possibly due to the smaller sample size of CovidLife.

Collectively, these results indicate that genetic liability to several psychiatric disorders and related traits, particularly INS, MD, and NEU, is associated with a more severe course of acute COVID-19, consistently observed across severity definitions based on time spent bedridden and symptom count. While the PGS for INS showed the most consistent associations across the two COVID-19 severity definitions and three models, the PGS for MD had the largest effect magnitudes.

### Psychiatric PGSs and non-COVID-19 acute respiratory illness severity

**Fig. 3:**
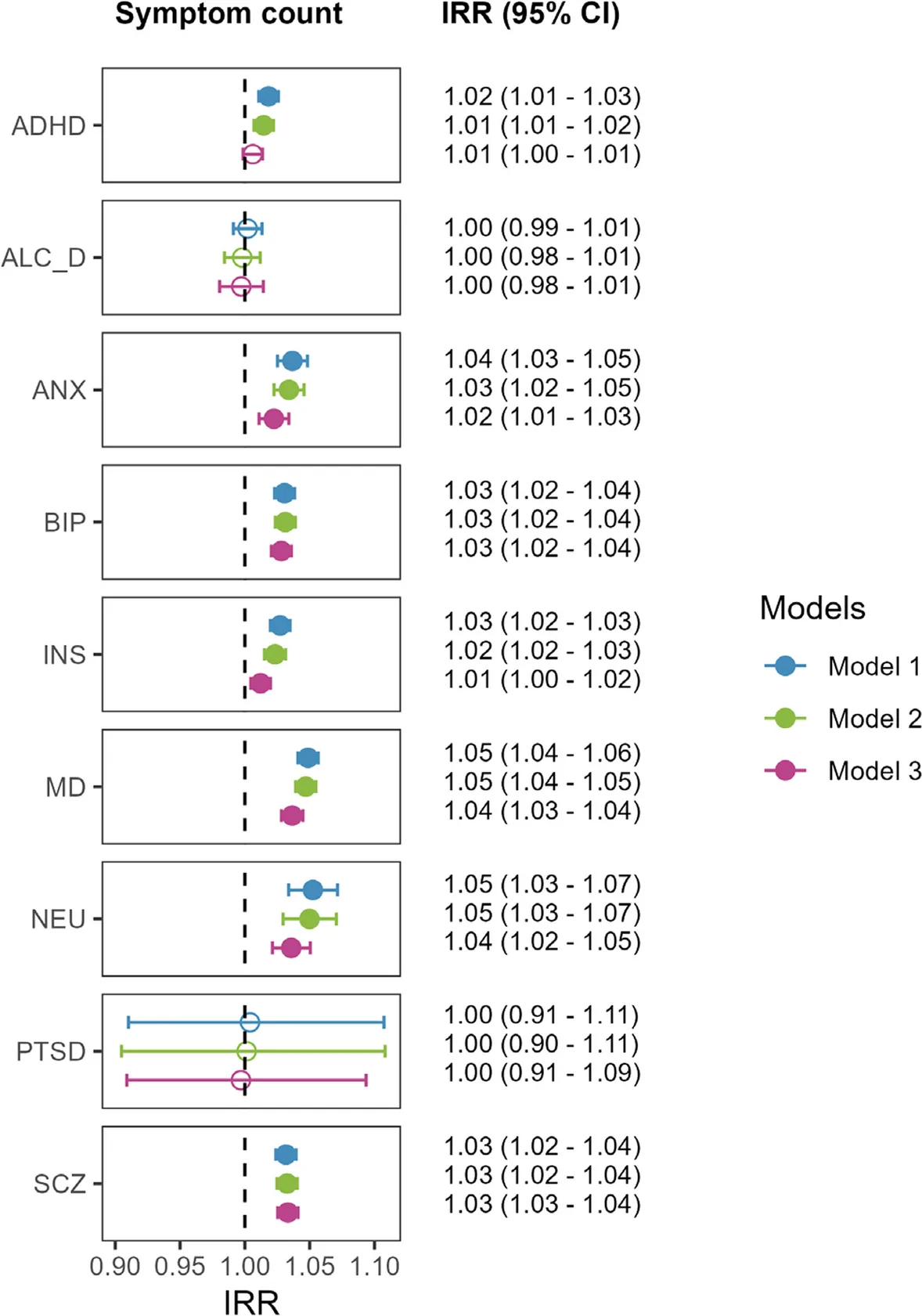
Meta-analysed incidence rate ratios of illness severity defined as symptom count for non-COVID-19 acute respiratory infection (max N = 46 319 in Model 1). Notes: Model 1: PGS + Age + Sex + 10 PCs; Model 2: Model 1 + Education + Smoking + BMI; Model 3: Model 2 + Previous psychiatric diagnosis + Somatic comorbidities + Response period + Time since testing. Cohorts included: the Icelandic COVID-19 National Resilience Cohort, the UK-based CovidLife study, the Danish Blood Donor Study, and the Estonian Biobank COVID-19 cohort. For N per phenotype, cohort and model, please refer to Additional file 1: Table S8. ADHD—attention-deficit/hyperactivity disorder, ALC_D—alcohol dependence, ANX—anxiety disorder, BIP—bipolar disorder, INS—insomnia, MD—major depression, NEU—neuroticism, PTSD—post-traumatic stress disorder, SCZ—schizophrenia. Filled circles—significant after Bonferroni correction for nine psychiatric PGSs (*p* < 0.0056). ARI—acute respiratory illness, RRR—meta-analysed relative risk ratio, IRR—meta-analysed incidence rate ratio, CI—confidence intervals

To test whether the associations observed with the psychiatric PGSs and illness severity were COVID-19 specific or apply also to other respiratory infections, we investigated the link between the psychiatric PGSs and symptom count in individuals with ARI symptoms who tested negative for SARS-CoV-2 (N = 46 319; Fig. 3). PGSs for seven psychiatric phenotypes associated with non-COVID-19 ARI severity defined as symptom count: ADHD, ANX, BIP, INS, MD, NEU, SCZ, with effect sizes ranging from 1.02–1.05 per 1 SD change in PGS in Model 1. The inclusion of education and lifestyle factors in Model 2 had very minimal effect on the estimates. In the fully adjusted Model 3, the association estimates were generally slightly attenuated, and the ADHD PGS became non-significant. Overall, PGSs for INS, MD, and NEU, previously associated with COVID-19 symptom count, were also associated with non-COVID-19 ARI symptoms, while PGSs for ANX, BIP, and SCZ showed associations only with non-COVID-19 ARI symptoms, independent of sociodemographic and lifestyle factors, psychiatric diagnoses, and somatic comorbidities. The *I*^2^ statistic indicated heterogeneity in the associations between PGS for ALC_D (Models 2 and 3, *I*^2^ = 58% and *I*^2^ = 58%, *p* < 0.05), NEU (Models 1 and 2, *I*^2^ = 73% and *I*^2^ = 77%, *p* < 0.05), and PTSD (across all models, *I*^2^ = 98–97%, *p* ≤ 0.001) with non-COVID-19 ARI severity. Excluding the CovidLife cohort had minimal impact on the heterogeneity estimates (Additional file 1: Table S9).

### Decile analysis of psychiatric PGSs and acute respiratory infection severity

**Fig. 4:**
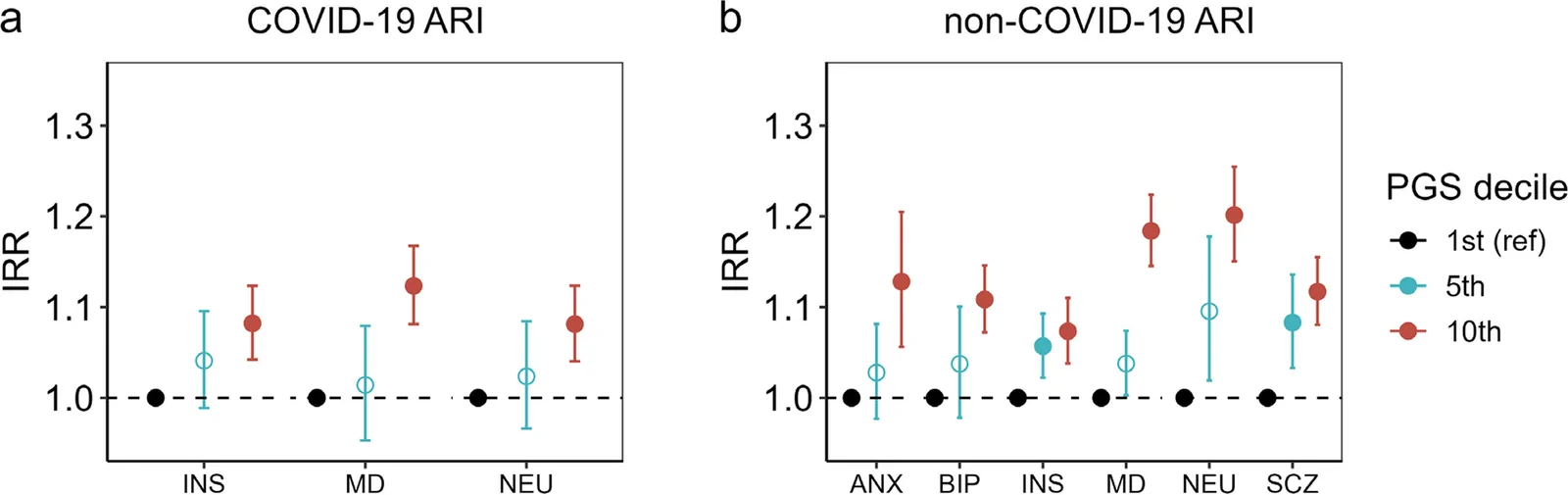
Meta-analysed estimates of ARI illness severity (symptom count) for **a** COVID-19 (N = 19 248) and **b** non-COVID-19 ARI (N = 45 673), based on contrasts between the 5th and 1st, and 10th and 1st deciles of the psychiatric polygenic scores (PGSs) that showed association with ARI illness severity after multiple testing correction in Model 1. Notes: Model 1: PGS + Age + Sex + 10 PCs. Cohorts included: **a** the Icelandic COVID-19 National Resilience Cohort, the Danish Blood Donor Study, the Estonian Biobank COVID-19 cohort, and the Norwegian Mother, Father, and Child Cohort study. **b** the Icelandic COVID-19 National Resilience Cohort, the Danish Blood Donor Study, and the Estonian Biobank COVID-19 cohort. Filled circles—significant after Bonferroni correction for **a**) six (*p* < 0.0083) or **b**) twelve tests (*p* < 0.0042), respectively. ANX—anxiety disorder, BIP—bipolar disorder, INS—insomnia, MD—major depression, NEU—neuroticism, SCZ—schizophrenia. ARI—acute respiratory illness, IRR—meta-analysed incidence rate ratios, CI—confidence intervals

To investigate the potential utility of genomics-based stratification to identify individuals in high risk for more severe ARI illness course, we compared the IRRs for symptom count of groups of individuals with the highest (PGS top 10%), average (PGS 50%) and lowest (PGS bottom 10%) genetic liability to psychiatric disorders and traits linked to ARI severity above: INS, MD, and NEU for COVID-19 and ANX, BIP, INS, MD, NEU, and SCZ for non-COVID-19 groups. The meta-analysed results indicate a dose–response pattern of the effects where the top decile group has consistently the highest incident rate ratio while the bottom decile group has the lowest (Fig. 4). Individuals in the 10th decile for MD and NEU PGS showed 20% increase in non-COVID-ARI symptom count compared to the 1st decile. Similarly, the 10th decile for all other psychiatric PGSs showed somewhat increased illness severity compared to the bottom decile. However, when contrasting the 10th and 5th deciles, the top decile did not differ statistically significantly in most analyses, indicating that the high-risk group (10th decile) was not distinguishable from the population average. The only exception was the PGS for MD, where the top decile differed statistically significantly from the 5th decile in both COVID-19 and non-COVID-19 groups.

### Somatic PGSs and acute respiratory illness severity

To compare the contributions of the psychiatric genetic liability to the genetic liability of known somatic risk factors for more severe respiratory illness course, we explored the effect estimates for the PGSs for IHD and T2D in both COVID-19 and non-COVID-19 ARI illness severity. Overall, the somatic PGSs (IHD, and T2D) did not significantly associate with COVID-19 illness severity as measured by days bedridden (Additional file 1: Fig. S2) nor symptom count (Additional file 1: Fig. S3) in any of the three models. We further explored the association between COVID-19 severity PGS (derived from a GWAS comparing hospitalized COVID-19 cases to the general population) and ARI severity. For time spent bedridden (contrasting 0 vs. more than 7 days), associations in Models 1 and 2 did not survive correction for multiple testing (Additional file 1: Fig. S2). On the other hand, we found that COVID-19 severity PGS predicted COVID-19 illness severity as measured by symptom count in Models 1 and 2 (IRR = 1.02 [1.01–1.03]; IRR = 1.02 [1.01–1.02], respectively; *I*^*2*^ 0%, NS) but did not associate with non-COVID-19 ARI, providing validation of the specificity of the COVID-19 severity PGS (Additional file 1: Fig. S3). However, after Model 3 covariate adjustments, this association reduced and did not survive correction for multiple testing. Overall, the contribution of the genetic liability of all three somatic phenotypes was weaker compared to the psychiatric genetic liability in predicting COVID-19 severity.

## Discussion

In this multicohort study including 78 507 individuals from community-based samples in five Northern European countries, we found that genetic liability to psychiatric disorders and related traits was associated with acute illness severity in COVID-19, measured as both days bedridden and symptom count, and with symptom count in non-COVID-19 conditions. These findings suggest that psychiatric genetic liability reflects a broader vulnerability to acute respiratory illness severity rather than effects specific to COVID-19, as similar patterns were observed across both COVID-19 and non-COVID-19 ARI. Overall, higher genetic liability to major depression, insomnia and neuroticism was consistently associated with more severe acute respiratory illness even when adjusting for socioeconomic and lifestyle factors, as well as psychiatric history and somatic comorbidities. Additionally, genetic liability for bipolar disorder, schizophrenia, anxiety disorder and attention-deficit/hyperactivity disorder demonstrated associations but less consistently across groups and models.

We demonstrate consistent associations between genetic liability to psychiatric disorders and neuroticism and ARI severity in the community setting where most individuals have not been hospitalized for severe ARIs. Our findings are in line with previous studies investigating the genetic link between psychiatric disorders and hospitalization and death due to COVID-19, showing evidence of a genetic link between stress-related disorders, substance misuse, depression and anxiety [14, 33]. In addition to COVID-19 severity, we also tested the associations with non-COVID-19 ARI severity and revealed similar effect sizes compared to COVID-19 severity. These results suggest that individuals with high genetic liability to psychiatric disorders and neuroticism are generally at greater risk of experiencing a more severe ARI course, including COVID-19.

We found that the genetic liability to MD showed the strongest and most consistent association with both COVID-19 and non-COVID-19 ARI severity across all analyses. Similar but slightly smaller associations were detected for genetic liability to insomnia and neuroticism. The rigorously adjusted models indicated that these associations cannot be entirely explained by mediation by education and BMI or pre-existing psychiatric diagnoses and somatic comorbidities. Hence, these results suggest shared genetic factors underlying mental health problems and vulnerability to more severe acute respiratory illnesses. Potential mechanistic avenues linking psychiatric outcomes with COVID-19 infection and hospitalization include immune-related pathways [34, 35]. Prior research has established links between depression and dysregulation of immune response [36]as well as hypothalamic–pituitary–adrenal axis hyperactivity [37], which may explain the detected associations on the genetic level. However, while previous studies have focused on diagnosed depression, here we detected increased vulnerability to ARI severity in individuals with high genetic predisposition to depression, regardless of existing depression diagnosis. These results highlight individuals with high genetic predisposition to depression as a vulnerable group for more severe ARI, which could be useful for future targeted prevention strategies in personalized health care systems, such as vaccine prioritization. However, considering the notable genetic overlap among psychiatric disorders [38–40] future studies should investigate the independent role of depression genetics in these associations.

Given the consistency across associations with MD, NEU and insomnia, we further explored their biological basis by reviewing the nearest genes to the top lead SNPs from their respective discovery GWASs. This revealed that associated loci predominantly implicate neuronal, synaptic, and brain-expressed regulatory mechanisms rather than direct immune-related pathways. This pattern suggests that the observed associations are more consistent with indirect pathways linking psychiatric genetic liability to infection outcomes, including neuroendocrine, behavioural, and systemic mechanisms such as stress-response dysregulation and sleep disturbance, which are known to influence immune function [41, 42]. Together, these findings support a model in which psychiatric genetic liability contributes to ARI severity primarily through centrally mediated and behavioural pathways rather than direct immune genetic effects.

In addition to the biologically mediated pathways, measurement-related factors should also be considered. Considering the somewhat subjective nature of the retrospective self-reported symptom assessments or days bedridden used in this study, it is possible that the illness course is perceived as more severe by individuals with higher genetic predisposition to these mental conditions, and some of the COVID-related questions could also be capturing symptoms/manifestations of depression, e.g. fatigue and being bedridden. However, the broadly consistent findings across both severity definitions suggest that reporting bias alone is unlikely to fully explain the observed associations, although it may contribute to them. The consistency of findings across both symptom count and days bedridden definitions, the latter being less dependent on individual symptom interpretation, further argues against symptom reporting bias as the sole explanation for the observed associations. Notably, adjustment for diagnosed psychiatric disorders had only minimal effects on the estimates, further suggesting that the observed associations are not solely attributable to psychiatric symptom overlap or reporting tendencies. Moreover, our findings are in line with previous studies using more objective illness severity indicators, such as COVID-19 hospitalization and mortality, which have also detected shared genetics with depression and other psychiatric conditions [14, 29], and stand adjustment for diagnosed psychiatric disorders. Therefore, the observed associations may reflect a combination of biological differences in illness severity and variation in symptom perception and reporting. Future studies investigating symptom-level overlap and whether psychiatric polygenic scores are associated with symptom reporting tendencies in non-infectious contexts may help disentangle biological susceptibility from potential reporting-related mechanisms.

We also found evidence for the genetic link between anxiety, bipolar disorder, schizophrenia and non-COVID-19 ARI. While associations with similar magnitudes were observed in the COVID-19 group, they did not remain significant after correction for multiple testing. This may result from the lower statistical power in the COVID-19 group, considering the smaller meta-analytical sample size in symptom count-based analysis of this group compared to non-COVID-19 ARI (ca 19 000 vs. 46 000, respectively). In addition, we did not detect notable associations with ADHD, alcohol dependence or PTSD polygenic liability. ADHD PGS was weakly associated with non-COVID-19 ARI severity in Models 1 and 2, but this association was attenuated after additional adjustments for psychiatric and somatic comorbidities in Model 3. This suggests that the increased vulnerability sometimes detected in ADHD patients may be linked to other mechanisms (e.g. comorbid health conditions) rather than shared genetic factors [43].

More generally, differences across psychiatric PGSs may also reflect variation in their predictive performance due to the variability in the discovery GWASs. The discovery GWASs included in our study differ in sample size and power to detect genetic signals, number of genome-wide significant loci detected, polygenicity, and SNP-based heritability (*h*^2^_SNP_), all of which are known to influence PGS performance. For example, traits such as major depression (N≈807 k, *h*^2^_SNP_≈0.09 [19]), insomnia (N≈1.3 M, *h*^2^_SNP_≈0.07 [20]), neuroticism (N≈450 k, *h*^2^_SNP_≈0.10 [18]), and schizophrenia (*h*^2^_SNP_≈0.24 [44]) are based on large GWASs with substantial numbers of associated variants, enabling more precise estimation of SNP effects. In contrast, phenotypes such as alcohol dependence (N≈39 k, *h*^2^_SNP_≈0.09 [45]) and PTSD (N≈175 k, *h*^2^_SNP_≈0.05 [46]) are derived from smaller GWASs with fewer genome-wide significant loci, potentially limiting PGS accuracy. Thus, given the different maturity levels of the PGSs, both GWAS scale and underlying genetic architecture jointly determine the signal captured by each PGS, and part of the heterogeneity observed across psychiatric phenotypes in our analyses may therefore reflect differences in PGS maturity rather than true absence of shared biological mechanisms. Taken together, observed differences likely reflect a combination of variation in PGS performance driven by GWAS characteristics and true differences in biological relevance to pathways underlying ARI severity.

We did not find associations between the polygenic liability for IHD or T2D and ARI severity in either ARI group. While previous reports using non-individual GWAS summary results-based methods like Mendelian randomization and genetic correlations have identified genetic associations between COVID-19 severity and the two somatic conditions [47–49], studies using individual-level genetic data and adjustment for potential confounders are scarce and contradicting. While one study detected an association between PGS for T2D and COVID-19 severity after adjusting for age, sex, and BMI [50], two other studies did not identify associations with PGSs for either T2D or IHD following these adjustments [51, 52]. In our analyses, the absence of associations for IHD and T2D contrasts with the more consistent findings observed for psychiatric phenotypes. While differences in PGS performance may contribute to this contrast, they are unlikely to fully account for it. The independent discovery GWASs for IHD (N≈407 k; 45 loci) and T2D (N≈405 k; 55 loci) are of comparable scale to those for some psychiatric traits, such as neuroticism, although smaller than the largest psychiatric GWASs. This suggests that the null findings for IHD and T2D are unlikely to be solely explained by limited GWAS power or PGS maturity. Taken together, these results suggest that psychiatric disorders and related traits may show greater overlap with pathways relevant to ARI severity than the somatic conditions examined, although differences in genetic architecture and PGS performance should be considered when interpreting cross-trait comparisons.

To move towards genomics-based patient stratification for improved detection of individuals with higher vulnerability for more severe ARI, we explored whether individuals in the high genetic liability group significantly differ in their ARI severity compared to the lowest and the population average. Generally, individuals with the highest psychiatric genetic liability experienced more severe ARI than those with the lowest genetic liability (1st PGS decile). The PGS for MD showed significant difference between the top and population average groups, especially in non-COVID-19 ARI. As PGSs capture genetic liability irrespective of the clinical manifestation, these results suggest potential future utility of PGS-based population stratification beyond diagnosed psychiatric illness for detecting vulnerable individuals, when genetic data become more widely available and integrated into health care systems. However, the other psychiatric PGSs did not detect significant differences between the top and population average groups, highlighting that the currently available psychiatric PGSs still lack sufficient precision for accurate stratification to identify high risk individuals in the general population. Thus, at present, psychiatric PGSs should be considered complementary rather than superior to established clinical information such as psychiatric history. This is in line with the current general status in psychiatric genetics [53–55], although rapid progress is being made in this direction through continuing large-scale genome-wide association meta-analysis efforts to increase precision. Future studies need to continue exploring the clinical utility of psychiatric PGSs in the context of somatic outcomes when more precise PGSs become available.

This study has several notable strengths, including its multi-national design, large meta-analytical sample size, use of two COVID-19 severity definitions and comprehensive adjustment for multiple potential confounders, such as education, lifestyle and psychiatric and somatic comorbidities. Additionally, the consistency of effect directions across most phenotypes contributes to the robustness of the findings, with minimal heterogeneity observed. However, certain limitations should be acknowledged. Our analyses were conducted among participants with SARS-CoV-2 testing and symptom data, and therefore reflect variation in illness severity within this tested population rather than infection risk or outcomes in the general population at large. ARI severity measures were based on retrospective self-report, which may be influenced by individual differences in symptom perception and recall. Although consistent findings across multiple severity definitions and adjustment models provide robustness of the results, potential role of reporting bias cannot be fully excluded and should be further explored in the future. The predictive power of polygenic scores may vary due to differences in heritability, polygenicity, and GWAS discoverability and power. In addition, the focus on European populations limits generalizability to populations with differing ancestral background. Therefore, future explorations are required using improved and more inclusive polygenic predictors once these become available. Also, the recruitment strategies and cohort sizes and health-related characteristics differ across the voluntary-based cohorts in the COVIDMENT consortium, and not all cohorts have complete covariate data. This may impact analytical precision and power, as well as affect generalizability to the entire populations.

## Conclusions

People with high genetic predisposition to psychiatric disorders and neuroticism may be at risk for a more severe acute course of respiratory viral infections, including COVID-19. These findings support the presence of shared genetic vulnerability linking acute respiratory illness severity and several psychiatric traits such as anxiety, bipolar disorder, insomnia, major depression, neuroticism, and schizophrenia. These results underscore the importance of broader acknowledgement of the role of psychiatric predisposition in acute respiratory infection severity within community samples.

## Supplementary Information

Additional file 1. This file contains the supplementary methods, supplementary figures S1—S3, and supplementary tables S1—S12.

## Data Availability

GWAS summary statistic files are publicly available and provided in respective publications (Supplementary Table S5). The individual-level data underlying this article were subject to ethical approval and cannot be shared publicly due to data protection laws in each participating country. Person-level data from DBDS needed to reproduce this study cannot be made publicly available due to confidentiality legislation. Furthermore, this study is based on pseudonymised registry data located on a secure platform at Statistics Denmark. Meta-data and programs are available from the authors upon reasonable request and with permission of the DBDS steering committee, the Ethical Committee, and the Danish Data Protection Agency. Enquiries about legal possibilities for accessing these data within DBDS, scripts/codes and further information should be addressed to the corresponding author.

## Abbreviations

ADHD: Attention-deficit/hyperactivity disorder
ALC_D: Alcohol dependence
ANX: Anxiety disorder
ARI: Acute respiratory illness
BIP: Bipolar disorder
BMI: Body mass index
C-19 Resilience: The Icelandic COVID-19 National Resilience Cohort
CI: Confidence interval
COVID-19: Coronavirus disease 2019
DBDS: The Danish Blood Donor Study
EstBB-C19: The Estonian Biobank COVID-19 cohort
GWAS: Genome-wide association study
*h*^2^_SNP_: Single nucleotide polymorphism (SNP)-based heritability
IHD: Ischaemic heart disease
INS: Insomnia
IRR: Incidence rate ratio
MD: Major depression
MoBa: The Norwegian Mother, Father, and Child Cohort study
NEU: Neuroticism
PC: Genetic principal component
PGS: Polygenic score
PTSD: Post-traumatic stress disorder
q-RT-PCR: Quantitative reverse transcription polymerase chain reaction
RRR: Relative risk ratio
SARS-CoV-2: Severe acute respiratory syndrome coronavirus 2
SNP: Single nucleotide polymorphism
SCZ: Schizophrenia
T2D: Type 2 diabetes mellitus
