# Supplementary material for: Psychiatric genetic liability is associated with the severity of COVID-19 and other acute respiratory infections: an observational study across five Northern European countries
Source: Genome Med. 2026 Jul 28;18:164. doi: 10.1186/s13073-026-01723-w (PMC13692891; doi:10.1186/s13073-026-01723-w)
Supplement: Supplementary file 1 [file 13073_2026_1723_MOESM1_ESM.pdf]

Psychiatric genetic liability is associated with the severity of COVID-19 and other acute respiratory infections: an observational study across five Northern European countries

Kadri Kõiv, Ragna Bugge Askeland, Lea Arregui Nordahl Christoffersen, Ingibjörg Magnúsdóttir, Daniel McCartney, Ole A. Andreassen, Thor Aspelund, Sofiya Babok, Mie Topholm Bruun, Archie Campbell, Elizabeth C. Corfield, DBDS Genetic Consortium, Maria Didriksen, Christian Erikstrup, Estonian Biobank Research Team, Arna Hauksdóttir, Jóhanna Jakobsdóttir, Bitten Aagaard Jensen, Isabella Loft, Reedik Mägi, Dorte Helenius Mikkelsen, Sisse Rye Ostrowski, Johanne Hagen Pettersen, Liam Quinn, Erik Sørensen, Edda Bjork Thordardottir, Yue Wang, Thomas Werge, Helga Ask, Ole Birger Vesterager Pedersen, Unnur Anna Valdimarsdóttir, Kelli Lehto

Supplementary Material

Contents

Supplementary Methods 2
- Genotyping and imputation methods and quality control 2
  - C-19 Resilience 2
  - CovidLife 2
  - Danish Blood Donor Biobank (DBDS) 2
  - Estonian Biobank (EstBB-C19) 2
  - Norwegian Mother, Father and Child Cohort study (MoBa) 3
- Polygenic scores 3

Supplementary Figures 4
- Fig. S1. 4
- Fig. S2 5
- Fig. S3. 5

Supplementary Tables 6
- Table S1. Ethical approvals for each cohort. 6
- Table S2. Background and sociodemographic characteristics by cohort. 7
- Table S3. Symptom count score by cohort. 10
- Table S4. Overview of available covariates included in statistical models by cohort. 11
- Table S5. Overview of the discovery GWA studies used for polygenic risk score computations. 12
- Table S6. PGS associations with time bedridden in COVID-19 respondents by cohort. 13
- Table S7. PGS associations with symptom count in COVID-19 respondents by cohort. 15
- Table S8. PGS associations with symptom count in non-COVID-19 ARI respondents by cohort. 17
- Table S9. Heterogeneity estimates as assessed by I2 statistic (%) and Cochran's Q test's p. 19
- Table S10. Addressing sample overlap between GWAS discovery cohorts and target datasets. 22
- Table S11. DBDS Genetic Consortium Banner Authors. 23
- Table S12. STROBE Statement. 24
- References to Supplementary Material. 27

Supplementary Methods

Genotyping and imputation methods and quality control

C-19 Resilience

In Iceland WGS data from 63,460 Icelanders participating in various disease projects at deCODE genetics was used as a reference panel. The samples were sequenced using standard TruSeq (Illumina) methodology to an average genome-wide coverage of 40×. SNPs and insertions and deletions (InDels) were identified, and their genotypes were called using joint calling with Graphtyper [1]. Identified high quality variants[1]were imputed into 173,025 chip-typed individuals using Illumina SNP arrays, and the chip-typed individuals were long-range phased [2].

CovidLife

Genotyping of the Generation Scotland samples was carried out by the Genetics Core Laboratory at the Edinburgh Clinical Research Facility, University of Edinburgh, Scotland and was funded by the Medical Research Council UK and the Wellcome Trust (Wellcome Trust Strategic Award "STratifying Resilience and Depression Longitudinally" (STRADL) Reference 104036/Z/14/Z).

The genotyping of the first 9863 samples used the Illumina HumanOmniExpressExome-8 v1.0 BeadChip and the remainder were genotyped using the Illumina HumanOmniExpressExome-8 v1.2 BeadChip, with Infinium chemistry for both. Genotyping quality control was performed using the following procedures: individuals with a call rate less than 98% were removed, as were SNPs with a call rate less than 98% or Hardy-Weinberg equilibrium p value less than 1 × 10–6. Mendelian errors, determined using relationships recorded in the pedigree, were removed by setting the individual-level genotypes at erroneous SNPs to missing. Ancestry outliers who were more than six standard deviations away from the mean, in a principal component analysis of GS:SFHS [3] merged with 1092 individuals from the 1000 Genomes Project [4], were excluded. A total of 20,032 individuals (8227 male participants and 11,805 female participants) passed all quality control thresholds. The number of genotyped autosomal SNPs that passed all quality control parameters was 604,858. Data was imputed using the Haplotype Research Consortium (HRC) dataset [5].

Danish Blood Donor Biobank (DBDS)

In DBDS, DNA from whole blood was genotyped at deCODE Ggenetics, using the Illumina Global Screening Array chips and longrange phased using Eagle2 [6]. QC was performed prior to imputation and included removal of sample duplicates, genotype rates lower than 0.98, and ancestry outliers (less than 90 [7]) and EIGENSOFT (v 6.0.1) [8]. Only variants with a genotype rate higher than 0.95, a MAF > 0.01, and a p-value < 10^(-6) for HWE were retained for downstream imputation. In addition, LD pruning using a window size of 100 markers shifting by 25 markers, removed half of every variant-pair with a genotypic r^2 > 0.1. The LD pruned markers were used to calculate heterozygosity, as well as identity by state and sex. Finally, samples with outlying heterozygosity (> 5 SD from the median), a sample from each pair of samples with an IBS>0.9, samples where reported sex did not match sex determined by genotype, and all A/T and C/G markers were removed. Imputation of non-genotyped SNPs was then performed at deCODE genetics, using an inhouse workflow [9], where graphtyper [1] formed the reference panel. The reference panel at deCODE Genetics, Icleand included a total of approximately 25,000 individuals of North-Western European ancestry, with around 8,500 Danish individuals sequenced as part of other studies.

Estonian Biobank (EstBB-C19)

Genotyping of DNA samples from the Estonian Biobank was done at the Core Genotyping Lab of the Institute of Genomics, University of Tartu using the Illumina Global Screening Arrays (GSAv1.0, GSAv2.0, and GSAv2.0_EST). Altogether 206,448 samples were genotyped and then PLINK format files were created using Illumina GenomeStudio v2.0.4. During the quality control all individuals with call-rate < 95% or mismatching sex that was defined based on the heterozygosity of the X chromosome and sex in the phenotype data, were excluded from the analysis. Variants were filtered by call rate < 95% and HWE p-value < 1e-4 (autosomal variants only). Variant positions were updated to Genome Reference Consortium Human Build 37 and all variants were changed to be from TOP strand using reference information provided by Dr Will Rayner from the University of Oxford (https://www.well.ox.ac.uk/~wrayner/strand/). After QC the dataset contained 202,910 samples for imputation. Before imputation variants with MAF<1% and Indels were removed. Prephasing was done using the Eagle v2.3 software [10](the number of conditioning haplotypes Eagle2 uses when phasing each

sample was set to: --Kpbwt=20000) and imputation was carried out using Beagle v.18May20.d20 [11,12] with an effective population size ne=20,000. As a reference, Estonian population specific imputation reference of 2297 WGS samples was used [13]. Further, EstBB samples were combined with the 1000 genomes phase 3 dataset for ancestry analysis. Genetic principal components were calculated using a subset of quality controlled and pruned genotyped SNPs. This was further used to identify and remove samples that deviated from the main cluster.

Norwegian Mother, Father and Child Cohort study (MoBa)

The Norwegian Mother, Father and Child Cohort Study (MoBa) is a population [14] based pregnancy cohort study conducted by the Norwegian Institute of Public Health. Participants were recruited from all over Norway from 1999-2008. The women consented to participation in 41% of the pregnancies. The cohort includes approximately 114.500 children, 95.200 mothers and 75.200 fathers. Genotyping of participants in MoBa has been conducted through multiple research projects, spanning several years. Different genotyping centres and arrays have been used with varying selection criteria, altogether 238,001 samples were genotyped in 24 genotyping batches. In total, 207,569 unique individuals and 6,981,748 autosomal SNPs passed the MoBaPsychGen pipeline. For further details about selection criteria and genotyping of MoBa see [15]

Polygenic scores

PRS-CS[16]applies a Bayesian regression framework to infer posterior effect sizes of single nucleotide polymorphisms (SNP) using previously published GWAS summary statistics and an external linkage disequilibrium reference panel based on 1000 Genomes Project European ancestry dataset. We used a common SNP-list across C-19 Resilience, CovidLife, EstBB, and MoBa to ensure comparability across cohorts (variant quality control thresholds for imputed genotypes included MAF>0.01, imputation INFO score >0.8, ambiguous SNPs removed), resulting in 5,372,203 SNPs in the final SNP list for PGS computation. The genotype data for DBDS became available in later stage of the study and thus were not included in the construction of the common SNP list. However, the common SNP list was used in DBDS for PGS scoring. In case of discovery GWAS and target sample overlap, either leave-one-out summary statistics were used, overlapping individuals were removed from analyses (Table S10), or these phenotypes were omitted in this cohort (in CovidLife: MD, SCZ; in DBDS: ANX, PTSD).

Supplementary Figures

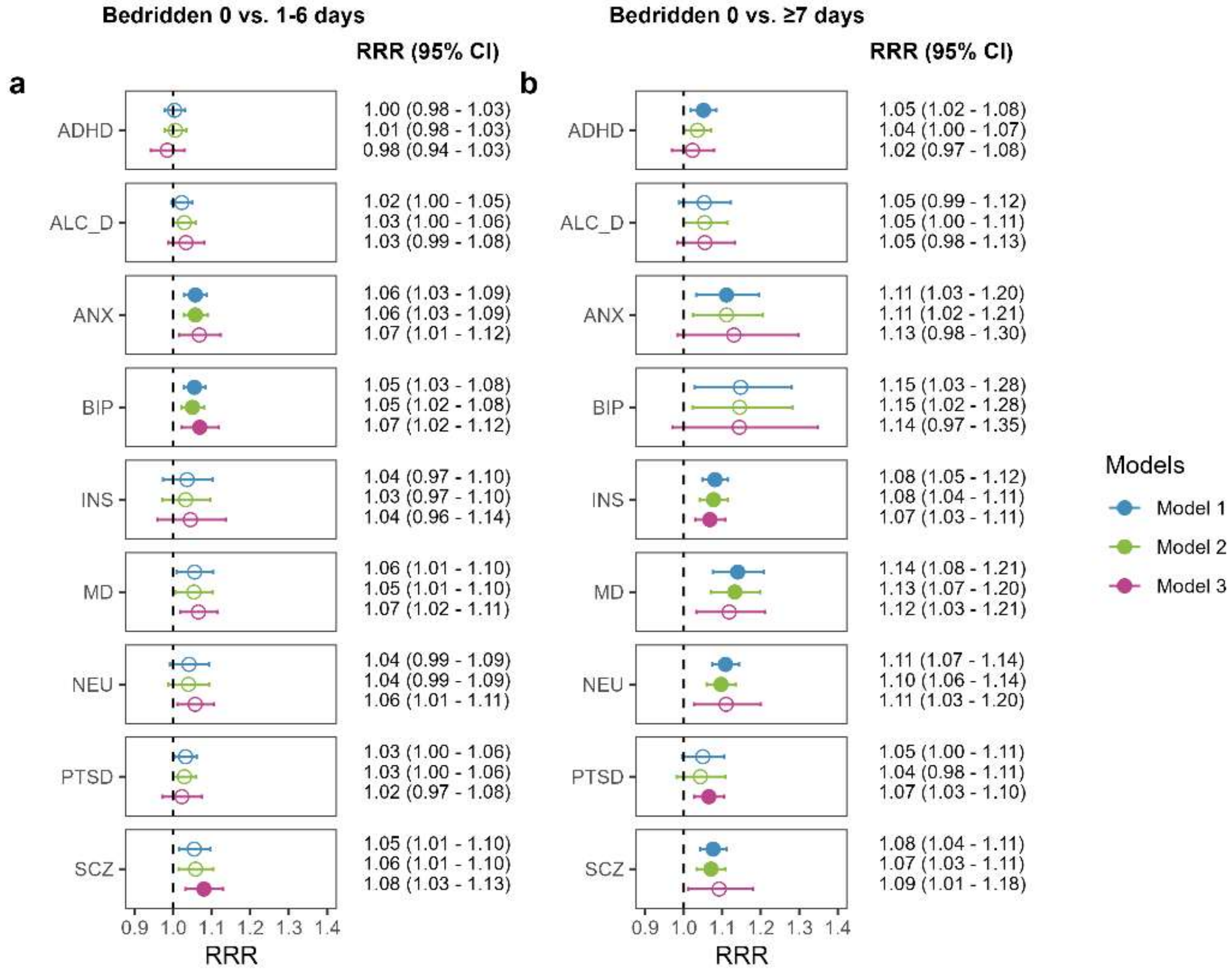

Fig. S1. Meta-analysed relative risk ratios for psychiatric PGS associations with COVID-19 severity status: a) bedridden 0 vs. 1-6 days (max N=13 855 and N=10 097 in Model 1, respectively) and b) bedridden 0 vs. 7+ days (max N= 13 855 and N=5 946 in Model 1, respectively).

Notes: Model 1: PGS + Age + Sex + 10 PCs; Model 2: Model 1 + Education + Smoking + BMI; Model 3: Model 2 + Previous psychiatric diagnosis + Somatic comorbidities + Response period + Time since testing. Cohorts included: a) the Icelandic COVID-19 National Resilience Cohort, the Danish Blood Donor Study, the Estonian Biobank COVID-19 Cohort, the Norwegian Mother, Father, and Child Cohort Study. b) the Icelandic COVID-19 National Resilience Cohort, the Estonian Biobank COVID-19 Cohort, the Norwegian Mother, Father, and Child Cohort Study. For N per phenotype, cohort and model, please refer to Table S6. ADHD - attention-deficit/hyperactivity disorder, ALC-D - alcohol dependence, ANX - anxiety disorder, BIP - bipolar disorder, INS - insomnia, MD - major depression, NEU - neuroticism, PTSD - post-traumatic stress disorder, SCZ - schizophrenia. Filled circles – significant after Bonferroni correction for nine psychiatric PGSs ($p<0.0056$). ARI - acute respiratory illness, RRR - meta-analysed relative risk ratio, CI - confidence intervals.

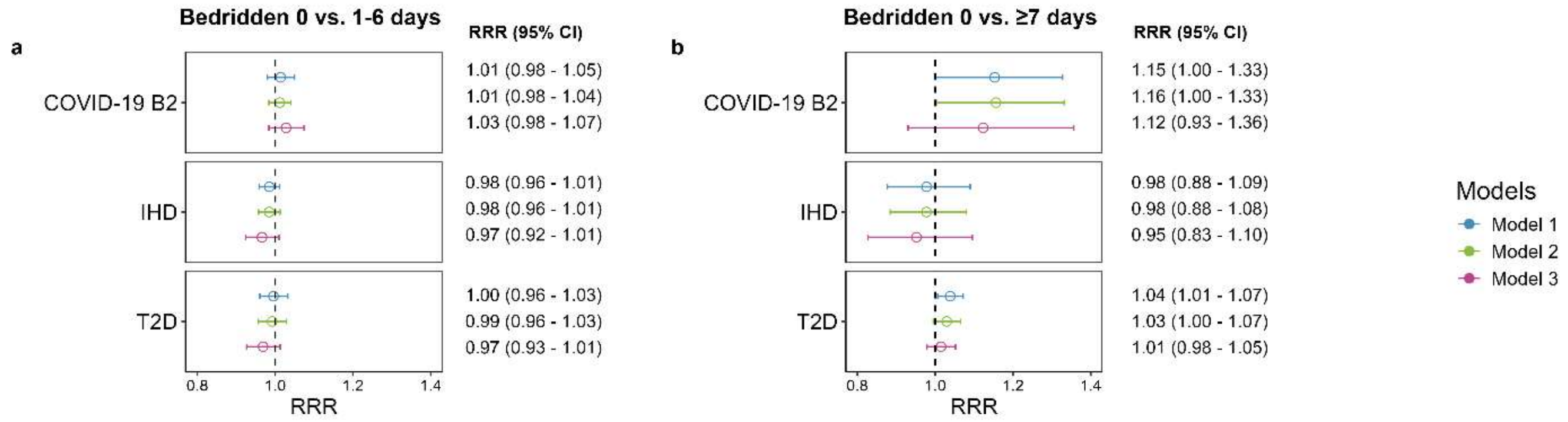

Fig. S2. Meta-analysed relative risk ratios for somatic PGS associations with COVID-19 severity status: a) bedridden 0 vs. 1-6 days (max N=13 855 and N=10 097 in Model 1, respectively) and b) bedridden 0 vs. 7+ days (max N= 13 855 and N=5 946 in Model 1, respectively).

Notes: Model 1: PGS + Age + Sex + 10 PCs; Model 2: Model 1 + Education + Smoking + BMI; Model 3: Model 2 + Previous psychiatric diagnosis + Somatic comorbidities + Response period + Time since testing. Cohorts included: a) the Icelandic COVID-19 National Resilience Cohort, the Danish Blood Donor Study, the Estonian Biobank COVID-19 Cohort, the Norwegian Mother, Father, and Child Cohort Study. b) the Icelandic COVID-19 National Resilience Cohort, the Estonian Biobank COVID-19 Cohort, the Norwegian Mother, Father, and Child Cohort Study. For N per phenotype, cohort and model, please refer to Table S6. ARI - acute respiratory illness, RRR - meta-analysed relative risk ratio, CI - confidence intervals. COVID-19 B2 - hospitalization due to COVID-19 vs. population, IHD - ischaemic heart disease. T2D - type 2 diabetes. Filled circles – significant after Bonferroni correction for three somatic PGSs ($p<0.017$).

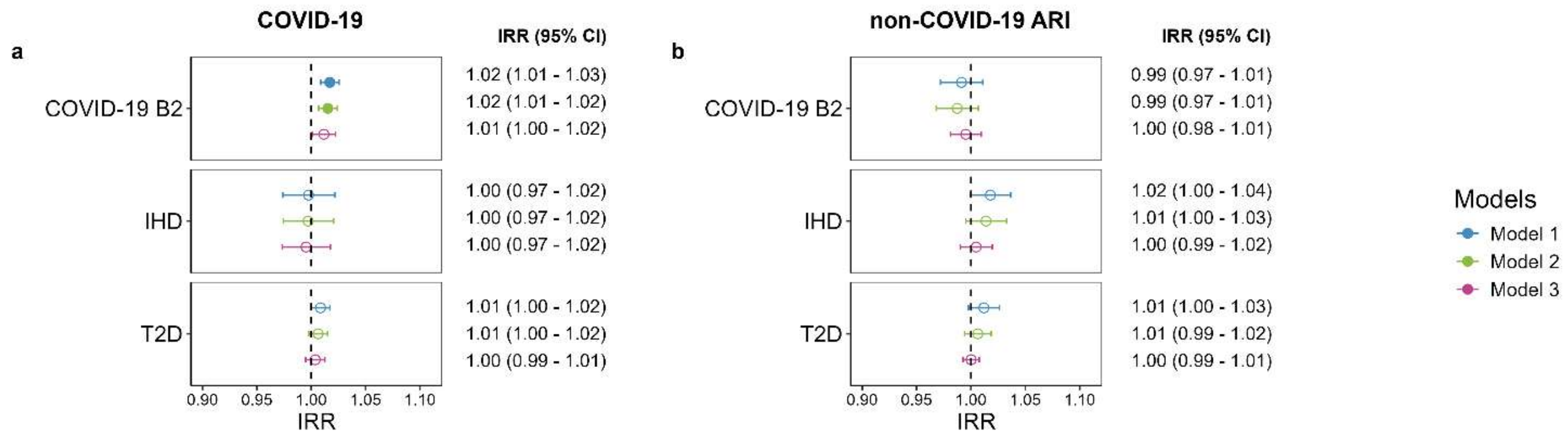

Fig. S3. Associations between somatic PGSs and (a) COVID-19 (max N=19 323 in Model 1) and (b) non-COVID-19 ARI symptom count (max N=46 319 in Model 1).

Notes: Model 1: PGS + Age + Sex + 10 PCs; Model 2: Model 1 + Education+ Smoking + BMI; Model 3: Model 2 + Previous psychiatric diagnosis + Somatic comorbidities + Response period + Time since testing. Cohorts included: a) the Icelandic COVID-19 National Resilience Cohort, the UK-based CovidLife study, the Danish Blood Donor Study, the Estonian Biobank COVID-19 cohort, the Norwegian Mother, Father, and Child Cohort study. b) the Icelandic COVID-19 National Resilience Cohort, the UK-based CovidLife study, the Danish Blood Donor Study, the Estonian Biobank COVID-19 cohort. For N per phenotype, cohort and model, please refer to Table S7. ARI = acute respiratory illness, IRR = meta-analysed incidence rate ratios, CI = confidence intervals. COVID-19 B2 - hospitalization due to COVID-19 vs. population, IHD - ischaemic heart disease, T2D - type 2 diabetes. Filled circles – significant after Bonferroni correction for three somatic PGSs ($p<0.017$).

Supplementary Tables

Table S1. Ethical approvals for each cohort.

**C19-Resilience**<br>Iceland	**CovidLife**<br>United Kingdom	**DBDS**<br>Denmark	**EstBB-C19**<br>Estonia	**MoBa**<br>Norway
National Bioethics Committee (NBC 20-073, 21–071) as well as the National Data Protection Authority.	Ethical approval for CovidLife is from the East of Scotland Research Ethics Service (EoSRES) ref 20/ES/0021/AM02.	DBDS was approved by the Danish Data Protection Agency (P-2019–99), as well as the Zealand (SJ-740), Central Denmark (1–10-72–95-13) and Capital Region of Denmark (H-21030945) Regional Committees on Health Research Ethics.	The activities of the EstBB are regulated by the Human Genes Research Act, which was adopted in 2000 specifically for the operations of the EstBB. Individual level data analysis in the EstBB was carried out under ethical approval [1.1-12/1277 and 1.1-12/212 25.01.2024] from the Estonian Committee on Bioethics and Human Research (Estonian Ministry of Social Affairs), using data according to release application [6-7/GI/17174] from the Estonian Biobank.	MoBa is regulated by the Norwegian Health Registry Act. This project was approved by The Regional Committees for Medical Research Ethics (REK) in 2016 (reference 2016/1702). Since then, the project has been amended and the amendment including COVID specific questions was approved by REK January 12th, 2022.

Table S2. Background and sociodemographic characteristics by cohort.

	C19-Resilience<br>Iceland<br>N=11 121		CovidLife<br>United Kingdom<br>N=721		DBDS<br>Denmark<br>N=33 186		EstBB C-19<br>Estonia<br>N=10 787		MoBa<br>Norway<br>N=22 692
	**COVID-19 N=5171**	**Non-COVID-19 ARI N=5950**	**COVID-19 N=75**	**Non-COVID-19 ARI N=646**	**COVID-19 N=1881**	**Non-COVID-19 ARI N=31 305**	**COVID-19 N=2369**	**Non-COVID-19 ARI N=8418**	**COVID-19 N=22 692**
**Gender**									
Male	1480 (28.6%)	1654 (27.8%)	23 (30.7%)	217 (33.6%)	867 (46.1%)	14814 (47.3%)	835 (35.2%)	2434 (28.9%)	7168 (31.6%)
Female	3691 (71.4%)	4296 (72.2%)	52 (69.3%)	429 (66.4%)	1014 (53.9%)	16491 (52.7%)	1534 (64.8%)	5984 (71.1%)	15524 (68.4%)
Other									
**Age**									
Mean age (SD)	56.5 (12.7)	55.5 (12.7)	54.2 (9.82)	56.8 (12.2)	47.6 (12.7)	51.4 (13.2)	44.4 (13.3)	44.8 (13.3)	45.6 (4.97)
Median [Min, Max]	58.0 [18.0, 93.0]	57.0 [19.0, 94.0]	56.0 [28.0, 77.0]	58.0 [27.0, 92.0]	48.3 [21.4, 77.3]*	52.8 [20.2, 83.9]*	43.0 [19.0, 86.0]	44.0 [19.0, 93.0]	45.0 [30.0, 74.0]
18-29 years	136 (2.6%)	224 (3.8%)	1 (1.3%)	8 (1.2%)	210 (11.2%)$	2252 (7.2%)	295 (12.5%)	1008 (12.0%)	-
30-39 years	419 (8.1%)	518 (8.7%)	4 (5.3%)	64 (9.9%)	355 (18.9%)	4762 (15.2%)	664 (28.0%)	2324 (27.6%)	2441 (10.8%)
40-49 years	911 (17.6%)	1043 (17.5%)	15 (20.0%)	98 (15.2%)	444 (23.6%)	6500 (20.8%)	625 (26.4%)	2099 (24.9%)	15661 (69.0%)
50-59 years	1464 (28.3%)	1650 (27.7%)	36 (48.0%)	186 (28.8%)	531 (28.2%)	8490 (27.1)	431 (18.2%)	1712 (20.3%)	4416 (19.5%)
60-69 years	1428 (27.6%)	1785 (30.0%)	17 (22.7%)	198 (30.7%)	269 (14.3%)$	7075 (22.6)	246 (10.4%)	929 (11.0%)	150 (0.7%)
70 years +	813 (15.7%)	730 (12.3%)	2 (2.7)	92 (14.2%)	52 (2.8%)$	2226 (7.1%)	108 (4.6%)	346 (4.1%)	5 (0.0%)
**Illness severity - symptom count**									
Mean symptom count (SD)	4.02 (2.22)	3.57 (2.24)	4.57 (2.53)	0.669 (1.83)	3.93 (2.79)	1.25 (2.11)	3.68 (2.81)	1.21 (2.01)	1.72 (1.07)
Median [Min, Max]	4.00 [0, 9.00]	4.00 [0, 9.00]	4.00 [0, 11.0]	0 [0, 11.0]	4.00 [0,10.0]*	0 [0,10.0]*	4.00 [0, 10.0]	0 [0, 10.0]	1.00 [1.00, 8.00]
Missing or not measured									12856 (56.7)
**Illness severity - bedridden**									
Never bedridden	1742 (33.7%)	NA		NA	960 (51.0%) &	NA	1199 (50.6%) £	NA	9962 (43.9%)
Bedridden 1-6 days	2947 (57.0%)	NA		NA	879 (46.7%) &&	NA	845 (35.7%) ££	NA	5431 (23.9%)
Bedridden 7 days or more	482 (9.3%)	NA		NA	NA	NA	325 (13.7%) £££	NA	5145 (22.7%)
Missing or not measured			75 (100%)		42 (2.2%)				2154 (9.5%)

	C19-Resilience		CovidLife		DBDS		EstBB C-19		MoBa
**Education**									
Compulsory	625 (12.1%)	761 (12.8%)	6 (8.0%)	42 (6.5%)	46 (4.2%)	1186 (3.8%)**	51 (2.2%)	154 (1.8%)	309 (1.4%)
Upper secondary, vocational or other	1460 (28.2%)	1794 (30.2%)	17 (22.7%)	153 (23.7%)	50 (2.7%)	821 (2.6%)	993 (41.9%)	3042 (36.1%)	5735 (25.3%)
Bachelor's/ diploma university degree	1812 (35.0%)	1983 (33.3%)	33 (44.0%)	273 (42.3%)	1207 (64.2%)$	20598 (65.8%)	654 (27.6%)	2358 (28.0%)	8434 (37.2%)
Master's or Ph.D.	1274 (24.6%)	1412 (23.7%)	18 (24.0%)	150 (23.2%)	571 (30.4%)	8700 (27.8%)	671 (28.3%)	2864 (34.0%)	7078 (31.2%)
No formal education			0 (0%)	10 (1.5%)					
Missing			1 (1.3%)	18 (2.8%)					1136 (5.0%)
**BMI (kg/m^2)**									
< 25, Normal weight	1425 (27.6%)	1672 (28.1%)	31 (41.3%)	250 (38.7%)	839 (44.6%)	14076 (45.0%)	1100 (46.4%)	3957 (47.0%)	9093 (40.1%)
25-30, Overweight	2026 (39.2%)	2326 (39.1%)	31(41.3%)	257 (39.8%)	717 (38.1%)	11982 (38.3%)	796 (33.6%)	2730 (32.4%)	6712 (29.6%)
> 30, Obese	1720 (33.3%)	1952 (32.8%)	13 (17.3%)	136 (21.1%)	325 (17.3%)	5247 (16.8%)	473 (20.0%)	1731 (20.6%)	3095 (13.6%)
Missing				3 (0.5%)					3792 (16.7%)
**Current smoking**									
No, never	2446 (47.3%)	2709 (45.5%)	44 (58.7%)	374 (57.9%)	1423 (75.7%)	23280 (74.4%)	1270 (53.6%)	4536 (53.9%)	18691 (82.4%)
No, former smoker	1948 (37.7%)	2367 (39.8%)	27 (36.0%)	220 (34.1%)	336 (17.9%)	5120 (16.4%)	803 (33.9%)	2687 (31.9%)	10 (0.0%)
Yes, currently	777 (15.0%)	874 (14.7%)	3 (4.0%)	45 (7.0%)	122 (6.5%)	2905 (9.3%)	296 (12.5%)	1195 (14.2%)	3960 (17.5%)
Missing			1 (1.3%)	7 (1.1%)					31 (0.1%)
**History of psychiatric disorders**									
No	3761 (72.7%)	4104 (69.0%)	54 (72.0%)	433 (67.0%)	1531 (81.4%)	25203 (80.5%)	1226 (51.8%)	4103 (48.7%)	19053 (84.0%)
Yes	1410 (27.3%)	1846 (31.0%)	21 (28.0%)	207 (32.0%)	350 (18.6%)	6102 (19.5%)	1143 (48.2%)	4315 (51.3%)	3639 (16.0%)
Missing				6 (0.9%)					
**Somatic comorbidities**									
No comorbidities	2984 (57.7%)	3328 (55.9%)	54 (72.0%)	436 (67.5%)	827 (44.0%)	12244 (39.1%)	1421 (60.0%)	4674 (55.5%)	
One comorbidity	1574 (30.4%)	1785 (30.0%)	14 (18.7%)	131 (20.3%)	628 (33.4%)	10918 (34.9%)	692 (29.2%)	2739 (32.5%)	186 (0.8%)
Two comorbidities	476 (9.2%)	631 (10.6%)	5 (6.7%)	43 (6.7%)	288 (15.3%)	5461 (17.4%)	187 (7.9%)	700 (8.3%)	22 (0.1%)
> Two comorbidities	137 (2.6%)	206 (3.5%)	2 (2.7%)	33 (5.1%)	138 (7.3%)	2682 (8.6%)	69 (2.9%)	305 (3.6%)	9 (0.0%)
Missing				3 (0.5%)					22475 (99.0%)

	C19-Resilience		CovidLife		DBDS		EstBB C-19		MoBa
**Response periods**									
April-June 2020	344 (6.7%)	2886 (48.5%)	10 (13.3%).	49 (7.6%)	0 (0%)	648 (2.1%)	161 (6.8%)	1180 (14.0%)	
July-September 2020	10 (0.2%)	44 (0.7%)	2 (2.7%)	132 (20.4%)	0 (0%)	27 (0.1%)	178 (7.5%)	1218 (14.5%)	
October-December 2020	125 (2.4%)	1793 (30.1%)	0 (0%)	0 (0%)	658 (35.0%)	13189 (42.1%)	191 (8.1%)	1374 (16.3%)	
January-March 2021	412 (8.0%)	431 (7.2%)	63 (84.0%)	465 (72.0%)	32 (1.7%)	444 (1.4%)	982 (41.5%)	2636 (31.3%)	
April-June 2021	39 (0.8%)	790 (13.3%)			1191 (63.3%)	16345 (52.2%)***	857 (36.2%) #	2010 (23.9%) #	
July-September 2021	2 (0.0%)	6 (0.1%)							
October-December 2021	1 (0.0%)	0 (0%)							
January-March 2022	-					0 (0%)			
April-June 2022	-					612 (2.0%)			
July-September 2022	2611 (50.5%)	0 (0%)				40 (0.1%)			
October-December 2022	1625 (31.4%)	0 (0%)							
January-March 2023	2 (0.0%)	0 (0%)							
Missing									22 692 (100 %)

Notes

* minimum and maximum was calculated as a mean of the five lowest and highest observations, respectively

** no formal education and compulsory is collapsed to avoid small numbers

***response period 5-7 collapsed to avoid small numbers

$ total has been calculated as a sum of Bedridden category 0 and 1

April-August 2021

Table S3. Symptom count score by cohort.

	**C19-Resilience**<br>Iceland	**CovidLife**<br>United Kingdom	**DBDS**<br>Denmark	**EstBB C-19**<br>Estonia	**MoBa**<br>Norway
**Symptoms**					
Cough	1	1	1	1	1
Difficulty breathing/Shortness of breath	1	1	1	1	1
Digestive problems/Stomach pain	1	1	1		
Fatigue/Weakness	1	1	1	1	1
Fever	1	1	1	1	1
Headache	1	1	1	1	1
Loss of/change in taste or smell	1	1	1	1	1
Muscle aches and/or joint/bone pains	1	1	1	1	1
Pneumonia		1		1	
Runny/constipated nose		1	1	1	
Sore throat	1	1	1	1	
Any other COVID-19 related symptom reported (0/1)				1	1
**Maximum score**	9	11	10	11	8

Table S4. Overview of available covariates included in statistical models by cohort.

		C19-Resilience	CovidLife	DBDS	EstBB-C19	MoBa
		Iceland	United Kingdom	Denmark	Estonia	Norway
Model 1	Age	×	×	×	×	×
	Gender	×	×	×	×	×
	10 PCs	×	×	×	×	×
Model 2	Education	×	×	×	×	×
	Smoking status	×	×	×	×	×
	Body mass index	×	×	×	×	×
Model 3	Previous psychiatric diagnosis*	×	×	Electronic health records: any ICD-10 F00-F99 diagnosis; or ICD-8 codes 295-200, 301-305, 307, 290-294, 309, 310, 315, 793, 980, 3080,3060, 3061, 3063, 3083, 3084, 3062, 3067, 3065, 3063, 3069; or medical register with the ATC codes: N05A, N06A, N05B.	Electronic health records: any ICD-10 F00-F99 diagnosis	×
	Number of chronic medical conditions**	×	×	×***	×	×****
	Response period	×	×	×	×	–
	Time since testing	×*****	×	×	×	–

Note: "×" denotes that covariate was included in models and "– "indicates that covariate was not included in models.

* Self-reported as yes or no if not stated differently

** Chronic conditions included are hypertension, diabetes, heart disease, lung disease, chronic kidney disease, cancer, or immunosuppressive therapy

*** Information on chronic kidney disease and immunosuppressive state or therapy not available in DBDS

**** A separate 'missing' category was used in MoBa to avoid dropping a large proportion of participants from the analysis

*****Information not available in C-19 Resilience in non-COVID-19 ARI group

Table S5. Overview of the discovery GWA studies used for polygenic risk score computations.

Psychiatric/somatic PGS	Discovery GWAS	N cases/controls	Independent GWAS hits (p<5x10-8)
**Attention deficit hyperactivity disorder (ADHD)** [17]	*Demontis et al. 2019*	20,183/35,191	12
**Alcohol dependence (ALC_D)** [18]	*Walters et al., 2018*	10,206/28,480	1
**Anxiety disorder (ANX)** [19]	*Purves et al., 2020*	25,453/58,113	5
**Bipolar disorder (BIP)** [20]	*Mullins et al., 2021*	41,917/371,549	64
**Insomnia (INS)** [21]	*Jansen et al.; 2019*	1,331,010	202
**Major depression (MD)** [22]	*Howard et al.; 2019*	246,363/561,190	102
**Neuroticism (NEU)** [23]	*Nagel et al., 2018*	449,484	136
**Post-traumatic stress disorder (PTSD)** [24]	*Nievergelt et al., 2019*	174,659	3
**Schizophrenia (SCZ)** [25]	*Trubetskoy et al., 2022*	53,386/77,258	287
**COVID-19 B2 phenotype (hospitalized vs population)** [26]	*COVID19 Host Genetics Initiative GWAS meta-analyses round 7*	44,986/2,356,386	40
**Ischemic heart disease (IHD)** [27,28]	*Phenotype code 411; UKBB*	31,952/ 374,656	45
**Type 2 diabetes (T2D)** [27,28]	*Phenotype code 250.2; UKBB*	18,105/387,087	55

Table S6. PGS associations with time bedridden in COVID-19 respondents by cohort**.** Relative risk ratios (RRRs) from multinomial logistic regression & 95% CIs. **Bold**: p<0.05

COVID-19 respondents	C19-Resilience (Iceland) N=5 171				DBDS (Denmark) N=1 839			
	Bedridden reference category	Model 1	Model 2	Model 3	Bedridden reference category	Model 1	Model 2	Model 3
Psychiatric PGS associations								
**ADHD**	0 vs. 1-6 days<br>0 vs. 7+ days	0.98 [0.93-1.04]<br>1.08 [0.98-1.20]	0.98 [0.92-1.04]<br>1.06 [0.96-1.17]	0.96 [0.91-1.03]<br>1.00 [0.61-1.62]	0 vs. 1-6 days<br>0 vs. 7+ days	1.04 [0.95-1.14]<br>NA	1.03 [0.93-1.13]<br>NA	1.02 [0.93-1.12]<br>NA
**ALC_D**	0 vs. 1-6 days<br>0 vs. 7+ days	1.03 [0.97-1.09]<br>1.04 [0.94-1.15]	1.03 [0.97-1.09]<br>1.03 [0.93-1.14]	1.02 [0.96-1.09]<br>1.00 [0.62-1.63]	0 vs. 1-6 days<br>0 vs. 7+ days	1.07 [0.98-1.18]<br>NA	1.07 [0.98-1.18]<br>NA	1.08 [0.98-1.18]<br>NA
**ANX**	0 vs. 1-6 days<br>0 vs. 7+ days	**1.08 [1.02-1.14]**<br>1.07 [0.97-1.19]	**1.08 [1.02-1.15]**<br>1.08 [0.97-1.19]	1.06 [1.00-1.12]<br>1.00 [0.62-1.63]	0 vs. 1-6 days<br>0 vs. 7+ days	NA<br>NA	NA<br>NA	NA<br>NA
**BIP**	0 vs. 1-6 days<br>0 vs. 7+ days	**1.06 [1.00-1.13]**<br>**1.12 [1.02-1.24]**	1.06 [1.00-1.13]<br>**1.13 [1.02-1.25]**	1.06 [1.00-1.13]<br>1.00 [0.62-1.62]	0 vs. 1-6 days<br>0 vs. 7+ days	1.09 [1.00-1.19]<br>NA	**1.11 [1.01-1.21]**<br>NA	**1.11 [1.01-1.21]**<br>NA
**INS**	0 vs. 1-6 days<br>0 vs. 7+ days	1.06 [1.00-1.12]<br>1.10 [0.99-1.21]	1.06 [1.00-1.12]<br>1.09 [0.99-1.21]	1.05 [0.98-1.11]<br>1.00 [0.62-1.62]	0 vs. 1-6 days<br>0 vs. 7+ days	**1.14 [1.04-1.26]**<br>NA	**1.14 [1.03-1.25]**<br>NA	**1.15 [1.04-1.26]**<br>NA
**MD**	0 vs. 1-6 days<br>0 vs. 7+ days	**1.09 [1.03-1.16]**<br>**1.19 [1.08-1.32]**	**1.09 [1.02-1.16]**<br>**1.18 [1.06-1.30]**	1.06 [0.99-1.12]<br>1.00 [0.61-1.62]	0 vs. 1-6 days<br>0 vs. 7+ days	**1.10 [1.00-1.21]**<br>NA	**1.11 [1.01-1.22]**<br>NA	**1.11 [1.01-1.22]**<br>NA
**NEU**	0 vs. 1-6 days<br>0 vs. 7+ days	1.06 [1.00-1.13]<br>1.09 [0.99-1.21]	1.06 [1.00-1.12]<br>1.08 [0.98-1.20]	1.04 [0.97-1.10]<br>1.00 [0.61-1.62]	0 vs. 1-6 days<br>0 vs. 7+ days	**1.13 [1.03-1.24]**<br>NA	**1.13 [1.03-1.24]**<br>NA	**1.12 [1.02-1.23]**<br>NA
**PTSD**	0 vs. 1-6 days<br>0 vs. 7+ days	1.04 [0.98-1.10]<br>0.99 [0.90-1.10]	1.03 [0.97-1.09]<br>0.98 [0.89-1.09]	1.01 [0.95-1.08]<br>1.00 [0.62-1.63]	0 vs. 1-6 days<br>0 vs. 7+ days	NA<br>NA	NA<br>NA	NA<br>NA
**SCZ**	0 vs. 1-6 days<br>0 vs. 7+ days	**1.07 [1.01-1.14]**<br>1.03 [0.93-1.14]	**1.07 [1.01-1.14]**<br>1.03 [0.93-1.15]	1.06 [1.00-1.13]<br>1.00 [0.62-1.63]	0 vs. 1-6 days<br>0 vs. 7+ days	1.09 [0.99-1.19]<br>NA	**1.11 [1.01-1.21]**<br>NA	**1.10 [1.01-1.21]**<br>NA
Somatic PGS associations								
**COVID-19 B2**	0 vs. 1-6 days<br>0 vs. 7+ days	1.02 [0.96-1.08]<br>**1.20 [1.09-1.33]**	1.02 [0.96-1.08]<br>**1.19 [1.08-1.32]**	1.01 [0.95-1.07]<br>1.00 [0.62-1.63]	0 vs. 1-6 days<br>0 vs. 7+ days	1.09 [1.00-1.20]<br>NA	1.09 [0.99-1.19]<br>NA	1.09 [0.99-1.20]<br>NA
**IHD**	0 vs. 1-6 days<br>0 vs. 7+ days	0.99 [0.94-1.05]<br>1.03 [0.93-1.14]	0.99 [0.94-1.06]<br>1.03 [0.93-1.13]	0.98 [0.92-1.04]<br>1.00 [0.62-1.63]	0 vs. 1-6 days<br>0 vs. 7+ days	0.93 [0.85-1.03]<br>NA	0.92 [0.84-1.01]<br>NA	0.92 [0.84-1.01]<br>NA
**T2D**	0 vs. 1-6 days<br>0 vs. 7+ days	0.97 [0.91-1.03]<br>1.07 [0.97-1.19]	0.97 [0.91-1.03]<br>1.07 [0.97-1.19]	0.97 [0.91-1.03]<br>1.00 [0.61-1.62]	0 vs. 1-6 days<br>0 vs. 7+ days	1.02 [0.93-1.12]<br>NA	1.01 [0.92-1.10]<br>NA	1.00 [0.91- 1.10]<br>NA

COVID-19 respondents	EstBB C-19 (Estonia) N=2 369					MoBa (Norway) N=20 519			
	N (if different)	Bedridden reference category	Model 1	Model 2	Model 3	Bedridden reference category	Model 1	Model 2	Model 3
Psychiatric PGS associations						N (if different)	20 519	17 190	17 174
**ADHD**		0 vs. 1-6 days	0.99 [0.91-1.08]	1.00 [0.91-1.09]	0.99 [0.91-1.09]	0 vs. 1-6 days	1.01 [0.97-1.04]	1.02 [0.98-1.05]	1.02 [0.98-1.05]
		0 vs. 7+ days	0.95 [0.84-1.07]	0.96 [0.85-1.08]	0.96 [0.85-1.09]	0 vs. 7+ days	**1.06 [1.02-1.09]**	**1.04 [1.00-1.08]**	1.04 [1.00-1.08]
**ALC_D**		0 vs. 1-6 days	1.00 [0.92-1.10]	1.01 [0.92-1.10]	1.01 [0.92-1.11]	0 vs. 1-6 days	1.02 [0.98-1.05]	1.03 [0.99-1.07]	1.03 [0.99-1.06]
		0 vs. 7+ days	0.97 [0.85-1.09]	0.98 [0.87-1.11]	0.99 [0.87-1.12]	0 vs. 7+ days	**1.09 [1.05-1.13]**	**1.08 [1.04-1.12]**	**1.08 [1.04-1.12]**
**ANX**		0 vs. 1-6 days	1.08 [0.99-1.18]	1.08 [0.99-1.18]	1.09 [0.99-1.20]	0 vs. 1-6 days	**1.05 [1.01-1.08]**	**1.05 [1.01-1.08]**	**1.04 [1.01-1.08]**
		0 vs. 7+ days	**1.24 [1.09-1.41]**	**1.25 [1.10-1.42]**	**1.26 [1.11-1.43]**	0 vs. 7+ days	**1.08 [1.04-1.12]**	**1.07 [1.03-1.11]**	**1.07 [1.03-1.11]**
**BIP**		0 vs. 1-6 days	1.05 [0.96-1.15]	1.05 [0.96-1.15]	1.05 [0.96-1.15]	0 vs. 1-6 days	**1.05 [1.01-1.08]**	1.04 [1.00-1.08]	1.04 [1.00-1.08]
		0 vs. 7+ days	**1.30 [1.15-1.48]**	**1.30 [1.15-1.47]**	**1.30 [1.14-1.47]**	0 vs. 7+ days	**1.07 [1.03-1.11]**	**1.06 [1.02-1.10]**	**1.06 [1.02-1.10]**
**INS**		0 vs. 1-6 days	0.97 [0.89-1.06]	0.97 [0.89-1.06]	0.97 [0.89-1.06]	0 vs. 1-6 days	1.00 [0.96-1.03]	0.99 [0.96-1.03]	0.99 [0.96-1.03]
		0 vs. 7+ days	1.07 [0.95-1.21]	1.08 [0.95-1.22]	1.08 [0.95-1.22]	0 vs. 7+ days	**1.08 [1.04-1.12]**	**1.08 [1.04-1.12]**	**1.07 [1.03-1.11]**
**MD**		0 vs. 1-6 days	1.05 [0.95-1.15]	1.04 [0.95-1.14]	1.05 [0.95-1.15]	0 vs. 1-6 days	1.02 [0.98-1.05]	1.01 [0.98-1.05]	1.01 [0.97-1.05]
		0 vs. 7+ days	**1.20 [1.05-1.36]**	**1.20 [1.05-1.36]**	**1.21 [1.06-1.38]**	0 vs. 7+ days	**1.11 [1.07-1.15]**	**1.10 [1.06-1.14]**	**1.09 [1.05-1.13]**
**NEU**	(N=2 273)	0 vs. 1-6 days	1.02 [0.93-1.11]	1.02 [0.93-1.12]	1.02 [0.93-1.12]	0 vs. 1-6 days	1.00 [0.97-1.04]	1.00 [0.96-1.03]	0.99 [0.96-1.03]
	(N=2 273)	0 vs. 7+ days	**1.18 [1.04-1.33]**	**1.19 [1.05-1.35]**	**1.20 [1.05-1.36]**	0 vs. 7+ days	**1.11 [1.07-1.14]**	**1.09 [1.05-1.13]**	**1.08 [1.04-1.13]**
**PTSD**		0 vs. 1-6 days	1.05 [0.96-1.15]	1.05 [0.96-1.15]	1.04 [0.95-1.14]	0 vs. 1-6 days	1.03 [0.99-1.06]	1.03 [0.99-1.06]	1.02 [0.99-1.06]
		0 vs. 7+ days	1.02 [0.90-1.16]	1.02 [0.90-1.16]	1.01 [0.89-1.15]	0 vs. 7+ days	**1.07 [1.04-1.11]**	**1.08 [1.04-1.12]**	**1.07 [1.03-1.11]**
**SCZ**	(N=2 311)	0 vs. 1-6 days	**1.10 [1.00-1.20]**	**1.10 [1.00-1.20]**	**1.10 [1.00-1.20]**	0 vs. 1-6 days	1.02 [0.99-1.06]	1.02 [0.98-1.06]	1.02 [0.98-1.06]
	(N=2 311)	0 vs. 7+ days	**1.17 [1.03-1.33]**	**1.18 [1.04-1.34]**	**1.18 [1.03-1.34]**	0 vs. 7+ days	**1.08 [1.04-1.11]**	**1.07 [1.03-1.11]**	**1.07 [1.03-1.11]**
Somatic PGS associations									
**COVID-19 B2**		0 vs. 1-6 days	1.01 [0.93-1.11]	1.01 [0.93-1.11]	1.01 [0.93-1.11]	0 vs. 1-6 days	0.99 [0.96-1.03]	1.00 [0.96-1.04]	1.00 [0.96-1.04]
		0 vs. 7+ days	**1.29 [1.15-1.44]**	**1.30 [1.16-1.46]**	**1.29 [1.15-1.46]**	0 vs. 7+ days	1.02 [0.98-1.05]	1.02 [0.99-1.06]	1.02 [0.99-1.06]
**IHD**		0 vs. 1-6 days	0.98 [0.89-1.07]	0.98 [0.90-1.07]	0.98 [0.90-1.08]	0 vs. 1-6 days	0.99 [0.96-1.02]	0.99 [0.96-1.03]	0.99 [0.95-1.03]
		0 vs. 7+ days	**0.86 [0.75-0.97]**	**0.86 [0.76-0.98]**	**0.86 [0.76-0.98]**	0 vs. 7+ days	1.03 [0.99-1.07]	1.02 [0.98-1.06]	1.02 [0.98-1.06]
**T2D**		0 vs. 1-6 days	0.95 [0.87-1.03]	0.95 [0.87-1.04]	0.95 [0.86-1.03]	0 vs. 1-6 days	1.02 [0.98-1.05]	1.02 [0.98-1.06]	1.02 [0.98-1.05]
		0 vs. 7+ days	0.99 [0.88-1.12]	0.99 [0.87-1.12]	0.98 [0.86-1.11]	0 vs. 7+ days	**1.04 [1.00-1.07]**	1.03 [0.99-1.07]	1.02 [0.98-1.06]

Table S7. PGS associations with symptom count in COVID-19 respondents by cohort. Incident rate ratios (IRRs) from Poisson's regression & 95% CIs. **Bold**: p<0.05

COVID-19 respondents	C19-Resilience (Iceland) N=5 171			CovidLife (UK) N=75			DBDS (Denmark) N=1 881		
	**Model 1**	**Model 2**	**Model 3**	**Model 1**	**Model 2**	**Model 3**	**Model 1**	**Model 2**	**Model 3**
Psychiatric PGS associations									
**ADHD**	1.01 [1.00-1.03]	1.00 [0.99-1.02]	1.00 [0.99-1.02]	1.04 [0.93-1.17]	1.12 [0.98-1.29]	**1.16 [1.01-1.34]**	1.02 [1.00-1.04]	1.02 [0.99-1.04]	1.01 [0.98-1.03]
**ALC_D**	1.00 [0.99-1.02]	1.00 [0.99-1.01]	1.00 [0.99-1.01]	1.01 [0.89-1.14]	1.01 [0.89-1.16]	0.93 [0.8-1.07]	1.00 [0.98-1.03]	1.01 [0.98-1.03]	1.01 [0.98-1.03]
**ANX**	**1.02 [1.00-1.03]**	**1.02 [1.00-1.03]**	1.01 [1.00-1.02]	1.14 [0.99-1.3]	1.14 [0.99-1.32]	1.06 [0.91-1.24]	NA	NA	NA
**BIP**	1.00 [0.99-1.02]	1.00 [0.99-1.02]	1.00 [0.98-1.01]	0.95 [0.81-1.11]	0.97 [0.81-1.16]	0.86 [0.7-1.05]	**1.04 [1.01-1.06]**	**1.04 [1.01-1.06]**	**1.03 [1.01-1.05]**
**INS**	**1.03 [1.01-1.04]**	**1.03 [1.01-1.04]**	**1.02 [1.00-1.03]**	1.02 [0.91-1.14]	1.04 [0.91-1.19]	1.01 [0.87-1.18]	1.02 [1.00-1.04]	1.02 [0.99-1.043]	1.02 [1.00-1.05]
**MD**	**1.04 [1.02-1.05]**	**1.03 [1.02-1.05]**	**1.02 [1.01-1.04]**	NA	NA	NA	**1.05 [1.03-1.08]**	**1.05 [1.03-1.08]**	**1.05 [1.02-1.07]**
**NEU**	**1.03 [1.02-1.05]**	**1.03 [1.01-1.04]**	**1.02 [1.00-1.03]**	0.96 [0.86-1.07]	0.95 [0.85-1.07]	0.92 [0.81-1.04]	**1.04 [1.01-1.06]**	**1.04 [1.01-1.06]**	**1.03 [1.00-1.05]**
**PTSD**	1.00 [0.98-1.01]	0.99 [0.98-1.01]	0.99 [0.98-1.01]	1.01 [0.88-1.16]	1.04 [0.89-1.22]	1.12 [0.94-1.35]	NA	NA	NA
**SCZ**	1.00 [0.99-1.02]	1.01 [0.99-1.02]	1.00 [0.99-1.02]	NA	NA	NA	**1.04 [1.02-1.07]**	**1.05 [1.02-1.07]**	**1.04 [1.01-1.06]**
Somatic PGS associations									
**COVID-19 B2**	**1.02 [1.00-1.03]**	1.01 [1.00-1.03]	1.01 [0.99-1.02]	1.03 [0.93-1.14]	1.03 [0.92-1.15]	1.04 [0.92-1.18]	**1.03 [1.01-1.05]**	**1.03 [1.01-1.05]**	**1.03 [1.00-1.05]**
**IHD**	1.01 [0.99-1.02]	1.01 [0.99-1.02]	1.00 [0.99-1.01]	0.95 [0.82-1.09]	0.93 [0.8-1.08]	0.91 [0.77-1.07]	1.01 [0.99-1.03]	1.01 [0.98-1.03]	1.01 [0.98-1.03]
**T2D**	1.01 [0.99-1.02]	1.01 [0.99-1.02]	1.00 [0.99-1.02]	1.05 [0.93-1.19]	1.07 [0.94-1.21]	0.99 [0.85-1.14]	1.01 [0.99-1.03]	1.01 [0.99-1.03]	1.01 [0.98-1.03]

COVID-19 respondents	EstBB C-19 (Estonia) N=2 369				MoBa (Norway) N=9 827			
	N (if different)	Model 1	Model 2	Model 3		Model 1	Model 2	Model 3
Psychiatric PGS associations					N (if different)	N=9 827	N=8 582	N=8 575
ADHD		0.99 [0.97-1.01]	0.99 [0.97-1.02]	0.99 [0.97-1.01]		**1.02 [1.01-1.04]**	1.01 [1.00-1.03]	1.01 [1.00-1.03]
ALC_D		1.01 [0.99-1.03]	1.01 [1.00-1.04]	1.01 [0.99-1.03]		1.01 [1.00-1.03]	1.01 [1.00-1.03]	1.01 [1.00-1.03]
ANX		**1.04 [1.02-1.07]**	**1.04 [1.02-1.06]**	**1.04 [1.02-1.07]**		1.01 [1.00-1.03]	1.01 [0.99-1.03]	1.01 [0.99-1.02]
BIP		**1.05 [1.03-1.07]**	**1.05 [1.03-1.07]**	**1.05 [1.02-1.07]**		1.01 [1.00-1.03]	1.02 [1.00-1.03]	1.02 [1.00-1.03]
INS		1.02 [1.00-1.04]	1.02 [1.00-1.04]	1.02 [0.99-1.04]		**1.03 [1.02-1.05]**	**1.03 [1.01-1.05]**	**1.03 [1.01-1.05]**
MD		**1.04 [1.02-1.06]**	**1.04 [1.01-1.06]**	**1.04 [1.01-1.06]**		**1.03 [1.01-1.04]**	**1.02 [1.01-1.04]**	**1.02 [1.00-1.04]**
NEU	N=2 273	1.01 [0.99- 1.04]	1.02 [0.99-1.04]	1.01 [0.99-1.04]		**1.03 [1.01-1.04]**	**1.03 [1.01-1.04]**	**1.02 [1.01-1.04]**
PTSD		**1.03 [1.01-1.05]**	**1.03 [1.01-1.05]**	1.02 [1.00-1.04]		1.01 [0.99-1.03]	1.01 [0.99-1.02]	1.01 [0.99-1.02]
SCZ	N=2 311	**1.07 [1.04-1.09]**	**1.07 [1.04-1.09]**	**1.06 [1.04-1.09]**		1.00 [0.99-1.02]	1.01 [0.99-1.02]	1.01 [0.99-1.02]
Somatic PGS associations								
COVID-19 B2		1.02 [1.00-1.04]	**1.02 [1.00-1.04]**	1.02 [1.00-1.04]		1.01 [1.00-1.03]	1.01 [0.99-1.03]	1.01 [0.99-1.03]
IHD		**0.96 [0.94-0.98]**	**0.96 [0.94-0.98]**	**0.96 [0.94-0.98]**		**1.02 [1.00-1.03]**	**1.02 [1.00-1.03]**	1.02 [1.00-1.03]
T2D		1.00 [0.98-1.02]	1.00 [0.98-1.02]	1.00 [0.98-1.02]		1.01 [1.00-1.03]	1.01 [0.99-1.02]	1.00 [0.99-1.02]

Table S8. PGS associations with symptom count in non-COVID-19 ARI respondents by cohort. Incident rate ratios (IRRs) from Poisson's regression & 95% CIs. **Bold**: p<0.05

**Non-COVID-19 ARI respondents**	**C19-Resilience (Iceland)** N=5 950			**CovidLife (UK)** N=646		
	**Model 1**	**Model 2**	**Model 3**	**Model 1**	**Model 2**	**Model 3**
Psychiatric PGS associations						
**ADHD**	**1.02 [1.00-1.03]**	1.01 [1.00-1.02]	1.00 [0.99-1.02]	**1.12 [1.02-1.24]**	1.05 [0.93-1.18]	1.03 [0.9-1.17]
**ALC_D**	1.01 [1.00-1.03]	1.01 [1.00-1.03]	1.01 [1.00-1.03]	1.04 [0.94-1.14]	**0.86 [0.77-0.96]**	**0.84 [0.75-0.95]**
**ANX**	**1.03 [1.02-1.05]**	**1.03 [1.02-1.05]**	**1.02 [1.01-1.04]**	1.05 [0.94-1.17]	1.02 [0.89-1.16]	1.04 [0.89-1.21]
**BIP**	**1.03 [1.02-1.05]**	**1.03 [1.02-1.04]**	**1.02 [1.01-1.04]**	**0.84 [0.74-0.95]**	0.99 [0.85-1.16]	1.01 [0.86-1.18]
**INS**	**1.03 [1.02-1.04]**	**1.02 [1.01-1.04]**	**1.01 [1.00-1.03]**	1.06 [0.96-1.17]	0.91 [0.82-1.01]	**0.88 [0.79-0.98]**
**MD**	**1.05 [1.04-1.07]**	**1.05 [1.03-1.06]**	**1.03 [1.02-1.05]**	NA	NA	NA
**NEU**	**1.05 [1.04-1.06]**	**1.05 [1.03-1.06]**	**1.04 [1.02-1.05]**	**1.13 [1.01-1.26]**	1.07 [0.93-1.23]	1.04 [0.9-1.21]
**PTSD**	**1.03 [1.01-1.04]**	**1.02 [1.01-1.04]**	**1.02 [1.00-1.03]**	**0.89 [0.8-0.99]**	**0.87 [0.77-0.99]**	**0.87 [0.76-0.99]**
**SCZ**	**1.03 [1.02-1.05]**	**1.03 [1.02-1.05]**	**1.03 [1.01-1.04]**	NA	NA	NA
Somatic PGS associations						
**COVID-19 B2**	1.01 [0.99-1.02]	1.01 [0.99-1.02]	1.01 [1.00-1.02]	1.05 [0.95-1.15]	0.96 [0.86-1.08]	0.96 [0.86-1.09]
**IHD**	1.01 [1.00-1.02]	1.01 [0.99-1.02]	1.00 [0.99-1.02]	1.04 [0.94-1.14]	0.97 [0.87-1.09]	0.97 [0.86-1.09]
**T2D**	**1.02 [1.00-1.03]**	1.01 [1.00-1.03]	1.00 [0.99-1.02]	**1.16 [1.05-1.29]**	1.03 [0.91-1.17]	1.04 [0.9-1.2]

Non-COVID-19 ARI respondents	DBDS (Denmark) N=31 305			EstBB C-19 (Estonia) N=8 418			
	**Model 1**	**Model 2**	**Model 3**	**N (if different)**	**Model 1**	**Model 2**	**Model 3**
Psychiatric PGS associations							
**ADHD**	**1.02 [1.01-1.03]**	**1.02 [1.01-1.03]**	1.01 [1.00-1.02]		1.02 [1.00-1.04]	1.01 [0.99-1.03]	1.00 [0.98-1.02]
**ALC_D**	1.00 [1.00-1.01]	1.00 [0.99-1.01]	1.00 [0.99-1.01]		0.99 [0.97-1.01]	0.99 [0.97-1.01]	0.98 [0.97-1.00]
**ANX**	NA	NA	NA		**1.04 [1.02-1.06]**	**1.04 [1.02-1.06]**	**1.02 [1.00-1.05]**
**BIP**	**1.03 [1.02-1.04]**	**1.03 [1.02-1.04]**	**1.03 [1.02-1.04]**		**1.04 [1.01-1.06]**	**1.03 [1.01-1.06]**	**1.03 [1.00-1.05]**
**INS**	**1.02 [1.01-1.03]**	**1.02 [1.01-1.03]**	1.01 [1.00-1.02]		**1.03 [1.01-1.05]**	**1.03 [1.01-1.05]**	**1.02 [1.00-1.04]**
**MD**	**1.05 [1.04 -1.06]**	**1.05 [1.04-1.06]**	**1.04 [1.03-1.05]**		**1.05 [1.03-1.07]**	**1.05 [1.02-1.07]**	**1.03 [1.01-1.05]**
**NEU**	**1.07 [1.06-1.08]**	**1.07 [1.06-1.08]**	**1.05 [1.04-1.06]**	N=8 016	**1.03 [1.01-1.05]**	**1.03 [1.01-1.05]**	1.02 [1.00-1.04]
**PTSD**	NA	NA	NA		**1.07 [1.05-1.09]**	**1.07 [1.05-1.09]**	**1.05 [1.03-1.08]**
**SCZ**	**1.03 [1.02-1.04]**	**1.03 [1.02-1.04]**	**1.04 [1.03-1.05]**	N=8 205	**1.05 [1.02-1.07]**	**1.05 [1.02-1.07]**	**1.04 [1.01-1.06]**
Somatic PGS associations							
**COVID-19 B2**	**0.97 [0.97-0.98]**	**0.97 [0.96-0.98]**	**0.99 [0.98-1.00]**		0.99 [0.97-1.01]	0.99 [0.97-1.01]	0.99 [0.97-1.01]
**IHD**	**1.03 [1.02-1.04]**	**1.03 [1.02-1.04]**	**1.02 [1.01-1.03]**		1.00 [0.98-1.02]	1.00 [0.98-1.02]	0.99 [0.97-1.01]
**T2D**	1.00 [0.99-1.01]	1.00 [0.99-1.01]	1.00 [0.99-1.01]		1.02 [1.00-1.04]	1.01 [0.99-1.03]	1.00 [0.98-1.02]

Table S9. Heterogeneity estimates as assessed by I^2 statistic (%) and Cochran's Q test's p. **Bold:** p<0.05

I^2 (%), Cochran's Q test's p-value	Bedridden in COVID-19 group							
	Bedridden 0 vs. 1-6 days				Bedridden 0 vs. 7 or more days			
	N of cohorts	Model 1	Model 2	Model 3	N of cohorts	Model 1	Model 2	Model 3
Psychiatric PGS associations								
**ADHD**	4	0, 0.77	0, 0.68	3.4, 0.55	3	0.26, 0.22	0, 0.41	11.2, 0.52
**ALC_D**	4	0, 0.73	0, 0.79	0, 0.78	3	46.1, 0.16	30.8, 0.26	24.6, 0.41
**ANX**	3*	0, 0.63	0, 0.57	0, 0.66	3	57.1, 0.11	63.0, 0.08	66.5, 0.05
**BIP**	4	0, 0.86	0, 0.60	0, 0.63	3	**79.8, <0.05**	**80.4, <0.01**	**76.0, <0.05**
**INS**	4	**74.2, <0.05**	**71.2, <0.05**	**73.2, <0.05**	3	0, 0.95	0, 0.97	0, 0.96
**MD**	4	50.1, 0.12	49.5, 0.11	31.2, 0.27	3	37.8, 0.24	32.8, 0.27	30.2, 0.32
**NEU**	4	56.7, 0.07	59.1, 0.06	51.0, 0.10	3	0, 0.62	0.09, 0.40	29.7, 0.33
**PTSD**	3*	0, 0.91	0, 0.91	0, 0.92	3	31.0, 0.29	40.9, 0.21	0, 0.70
**SCZ**	4	35.6, 0.25	42.9, 0.17	39.7, 0.20	3	0.04, 0.30	0.07, 0.28	28.0, 0.36
Somatic PGS associations								
**COVID-19 B2**	4	25.1, 0.26	0.29, 0.41	0.59, 0.42	3	**88.8, < .0001**	**88.4, < .0001**	**83.6, <0.001**
**IHD**	4	0, 0.70	0, 0.53	0, 0.60	3	**79.7, <0.05**	**75.2, <0.05**	**68.0, <0.05**
**T2D**	4	29.4, 0.26	27.07, 0.31	26.4, 0.31	3	0, 0.61	0, 0.58	0, 0.83

Cohorts: C-19 Resilience, DBDS, EstBB-C19, MoBa

Cohorts: C-19 Resilience, EstBB-C19, MoBa

*ANX & PTSD unavailable in DBDS

I^2 (%), Cochran's Q test's p-value	Symptom count in COVID-19 group							
	All cohorts available				Without the CovidLife cohort			
	N of cohorts	Model 1	Model 2	Model 3	N of cohorts without CovidLife	Model 1 without CovidLife	Model 2 without CovidLife	Model 3 without CovidLife
Psychiatric PGS associations								
**ADHD**	5	29.0, 0.27	0.4, 0.26	1.2, 0.17	4	37.8, 0.18	0, 0.4522	0, 0.4893
**ALC_D**	5	0, 0.91	0, 0.72	0.6, 0.60	4	0, 0.80	0, 0.5507	0, 0.6920
**ANX**	4*	55.2, 0.07	59.4, 0.05	66.8, 0.06	3	61.0, 0.10	65.44, 0.07	**75.5, <0.05**
**BIP**	5	**74.5, <0.01**	**71.6, <0.01**	**73.8, <0.01**	4	**79.8, <0.01**	**77.4, 0.01**	**78.7, <0.01**
**INS**	5	0, 0.80	0, 0.84	0, 0.88	4	0, 0.65	0, 0.72	0, 0.77
**MD**	4**	0, 0.40	17.6, 0.26	33.1, 0.20	4**	0, 0.40	17.6, 0.26	33.1, 0.20
**NEU**	5	2.0, 0.39	0.1, 0.49	5.3, 0.47	4	0, 0.48	0, 0.62	0, 0.88
**PTSD**	4*	58.8, 0.11	**66.9, <0.05**	44.4, 0.12	3	**69.1, <0.05**	**75.5, <0.05**	52.8, 0.12
**SCZ**	4**	**89.5, <0.0001**	**88.9, <0.0001**	**87.2, <0.001**	4**	**89.5, <0.0001**	**88.9, <0.0001**	**87.2, <0.001**
Somatic PGS associations								
**COVID-19 B2**	5	0, 0.88	0, 0.66	24.7, 0.34	4	0, 0.61	0, 0.50	30.0, 0.24
**IHD**	5	**83.4, <0.001**	**81.20, <0.01**	**79.4, <0.01**	4	**87.1, <0.001**	**85.2, <0.001**	**83.5, <0.01**
**T2D**	5	0, 0.88	0, 0.858	0, 0.96	4	0, 0.88	0, 0.92	0, 0.91

Cohorts: C-19 Resilience, CovidLife, DBDS, EstBB-C19, MoBa

*ANX & PTSD unavailable in DBDS

**MD & SCZ unavailable in CovidLife

I^2 (%), Cochran's Q test's p-value	Symptom count in non-COVID-19 ARI group							
	All cohorts available				Without the CovidLife cohort			
	N of cohorts	Model 1	Model 2	Model 3	N of cohorts without CovidLife	Model 1	Model 2	Model 3
Psychiatric PGS associations								
**ADHD**	4	0.60, 0.24	0, 0.7762	0.00, 0.94	3	0, 0.92	0, 0.67	0, 0.8602
**ALC_D**	4	39.5, 0.20	**57.7, <0.05**	**70.4, p<0.01**	3	52.4, 0.13	55.3, 0.11	64.53, 0.0824
**ANX**	3*	0, 0.89	0, 0.83	0, 0.94	2	0, 0.67	0, 0.581	0, 0.7809
**BIP**	4	0, 0.01	0, 0.95	0, 0.86	3	0, 0.87	0, 0.94	0, 0.7085
**INS**	4	0, 0.82	6.7, 0.15	0.61, 0.05	3	0, 0.77	0, 0.84	0, 0.43
**MD**	3**	0, 0.84	0, 0.98	8.5, 0.39	3**	0, 0.84	0, 0.98	8.5, 0.39
**NEU**	4	**73.0, <0.05**	**76.9, <0.01**	57.6, 0.12	3	**80.1, <0.01**	**83.9, 0.01**	68.1, 0.06
**PTSD**	3*	**97.9, <0.0001**	**97.9, <0.0001**	**97.4, <0.01**	2	**90.8, <0.01**	**91.7, <0.001**	**86.6, <0.01**
**SCZ**	3**	0.69, 0.42	0, 0.53	0.00, 0.57	3**	0.69, 0.42	0, 0.53	0.00, 0.57
Somatic PGS associations								
**COVID-19 B2**	4	**77.7,< 0.01**	**77.3,< 0.01**	58.6, 0.07	3	**83.3, <0.001**	**84.2, <0.001**	**68.1, <0.05**
**IHD**	4	**74.4, <0.01**	**75.2, <0.01**	61.1, 0.07	3	**82.2, <0.01**	**81.9, <0.01**	**70.0, <0.05**
**T2D**	4	**58.6, <0.01**	46.7, 0.19	0.00, 0.77	3	57.9, 0.09	56.2, 0.10	0, 0.6485

Cohorts: C-19 Resilience, CovidLife, DBDS, EstBB-C19

*ANX & PTSD unavailable in DBDS

**MD & SCZ unavailable in CovidLife

Table S10. Addressing sample overlap between GWAS discovery cohorts and target datasets.

	Strategies used to address sample overlap by cohort					*Number of SNPs in PRSs*	
Psychiatric PRS phenotypes	*C-19 Resilience*	*CovidLife*	*DBDS*	*EstBB-C19*	*MoBa*	*EstBB, CovidLife, MoBa, C-19 Resilience**	*DBDS*
**ADHD**			1			985 376	926 496
**ALC_D**						991 549	942 167
**ANX**			NA			1 008 433	NA
**BIP**	1 (1 008 387)		1	1 (1 008 390)		1 008 390	958 199
**INS**						987 105	938 037
**MD**		NA	1			995 709	957 667
**NEU**				2		987 138	938 044
**PTSD**			NA			1 008 301	NA
**SCZ**		NA	1	2		1 008 436	958 215
Somatic PRS phenotypes							
**COVID B2 hospitalization vs population**	1 (1 008 095)	1 (1 008 075)		1 (1 008 084)		1 008 084	958 174
**IHD**						1 007 434	957 450
**T2D**						1 007 434	957 450

Notes:

1 - leave-one-out summary statistics used (no of SNPs); DBDS used leave-one-out summary statistics without the IPsych cohort

2 - individuals part of the original GWAS removed from the analysis

* - common quality controlled SNPs used, please see Supplementary Methods section

NA - phenotype not available due to sample overlap

Table S11. DBDS Genetic Consortium Banner Authors.

n	First name	Surname	Affiliation 1	Affiliation 2
1	Karina	Banasik	Department of Obstetrics and Gynaecology, Copenhagen Univesity Hospital, Hvidovre Hospital, Copenhagen, Denmark	
2	Jakob	Bay	Department of Clinical Immunology, Zealand University Hospital, Køge, Denmark	
3	Andrea	Barghetti	Department of Clinical Immunology, Copenhagen University Hospital, Rigshospitalet, Copenhagen, Denmark	
4	Mette Skou	Bendtsen	Department of Clinical Immunology, Copenhagen University Hospital, Rigshospitalet, Copenhagen, Denmark	
5	Jens Kjærgaard	Boldsen	Department of Clinical Immunology, Aarhus University Hospital, Aarhus, Denmark	
6	Søren	Brunak	Novo Nordisk Foundation Center for Protein Research, Faculty of Health and Medical Sciences, University of Copenhagen, Copenhagen, Denmark	
7	Nanna	Brøns	Department of Clinical Immunology, Copenhagen University Hospital, Rigshospitalet, Copenhagen, Denmark	
8	Alfonso	Buil Demur	Institute of Biological Psychiatry, Mental Health Centre, Sct. Hans, Copenhagen University Hospital, Roskilde, Denmark	
9	Johan Skov	Bundgaard	Department of Clinical Immunology, Copenhagen University Hospital, Rigshospitalet, Copenhagen, Denmark	
10	Lea Arregui Nordahl	Christoffersen	Department of Clinical Immunology, Zealand University Hospital, Køge, Denmark	
11	Maria	Didriksen	Department of Clinical Immunology, Copenhagen University Hospital, Rigshospitalet, Copenhagen, Denmark	
12	Khoa Manh	Dinh	Department of Clinical Immunology, Aarhus University Hospital, Aarhus, Denmark	
13	Joseph	Dowsett	Department of Clinical Immunology, Copenhagen University Hospital, Rigshospitalet, Copenhagen, Denmark	
14	Christian	Erikstrup	Department of Clinical Immunology, Aarhus University Hospital, Aarhus, Denmark	Department of Clinical Medicine, Health, Aarhus University, Aarhus, Denmark
15	Josephine	Gladov	Department of Clinical Immunology, Aarhus University Hospital, Aarhus, Denmark	Department of Clinical Medicine, Health, Aarhus University, Aarhus, Denmark
16	Daniel	Gudbjartsson	deCODE Genetics, Reykjavik, Iceland	
17	Thomas Folkmann	Hansen	Danish Headache Center, Department of Neurology, Copenhagen University Hospital, Rigshospitalet-Glostrup, Copenhagen, Denmark	
18	Dorte	Helenius Mikkelsen	Institute of Biological Psychiatry, Mental Health Centre, Sct. Hans, Copenhagen University Hospital, Roskilde, Denmark	
19	Lotte	Hindhede	Department of Clinical Immunology, Aarhus University Hospital, Aarhus, Denmark	
20	Henrik	Hjalgrim	Danish Cancer Society Research Center, Copenhagen, Denmark	Department of Epidemiology Research, Statens Serum Institut, Copenhagen, Denmark
21	Jakob	Hjorth von Stemann	Department of Clinical Immunology, Copenhagen University Hospital, Rigshospitalet, Copenhagen, Denmark	
22	Bitten Aagaard	Jensen	Department of Clinical Immunology, Aalborg University Hospital, Aalborg, Denmark	
23	Kathrine	Kaspersen	Department of Clinical Immunology, Aarhus University Hospital, Aarhus, Denmark	
24	Bertram Dalskov	Kjerulff	Department of Clinical Immunology, Aarhus University Hospital, Aarhus, Denmark	
25	Lisette	Kogelman	Danish Headache Center, Department of Neurology, Copenhagen University Hospital, Rigshospitalet-Glostrup, Copenhagen, Denmark	
26	Mette	Kongstad	Department of Clinical Immunology, Copenhagen University Hospital, Rigshospitalet, Copenhagen, Denmark	
27	Susan	Mikkelsen	Department of Clinical Immunology, Aarhus University Hospital, Aarhus, Denmark	
28	Christina	Mikkelsen	Department of Clinical Immunology, Copenhagen University Hospital, Rigshospitalet, Copenhagen, Denmark	
29	Line Hjorth Sjernholm	Nielsen	Department of Clinical Immunology, Aarhus University Hospital, Aarhus, Denmark	Department of Clinical Medicine, Health, Aarhus University, Aarhus, Denmark
30	Janna	Nissen	Department of Clinical Immunology, Copenhagen University Hospital, Rigshospitalet, Copenhagen, Denmark	
31	Mette	Nyegaard	Department of Health Science and Technology, Faculty of Medicine, Aalborg Univeristy, Aalborg, Denmark	
32	Sisse Rye	Ostrowski	Department of Clinical Immunology, Copenhagen University Hospital, Rigshospitalet, Copenhagen, Denmark	Department of Clinical Medicine, Faculty of Health and Medical Sciences, University of Copenhagen, Copenhagen, Denmark
33	Frederikke Byron	Pedersen	Department of Clinical Immunology, Copenhagen University Hospital, Rigshospitalet, Copenhagen, Denmark	
34	Ole Birger	Pedersen	Department of Clinical Immunology, Zealand University Hospital, Køge, Denmark	Department of Clinical Medicine, Faculty of Health and Medical Sciences, University of Copenhagen, Copenhagen, Denmark
35	Liam James Elgaard	Quinn	Department of Clinical Immunology, Zealand University Hospital, Køge, Denmark	
36	Þórunn	Rafnar	deCODE Genetics, Reykjavik, Iceland	
37	Palle Duun	Rohde	Department of Health Science and Technology, Faculty of Medicine, Aalborg Univeristy, Aalborg, Denmark	
38	Klaus	Rostgaard	Danish Cancer Society Research Center, Copenhagen, Denmark	Department of Epidemiology Research, Statens Serum Institut, Copenhagen, Denmark
39	Andrew Joseph	Schork	Institute of Biological Psychiatry, Mental Health Centre, Sct. Hans, Copenhagen University Hospital, Roskilde, Denmark	
40	Michael	Schwinn	Department of Clinical Immunology, Copenhagen University Hospital, Rigshospitalet, Copenhagen, Denmark	
41	Erik	Sørensen	Department of Clinical Immunology, Copenhagen University Hospital, Rigshospitalet, Copenhagen, Denmark	
42	Kari	Stefansson	deCODE Genetics, Reykjavik, Iceland	
43	Hreinn	Stefánsson	deCODE Genetics, Reykjavik, Iceland	
44	Jacob	Træholt	Department of Clinical Immunology, Copenhagen University Hospital, Rigshospitalet, Copenhagen, Denmark	
45	Unnur	Þorsteinsdóttir	deCODE Genetics, Reykjavik, Iceland	
46	Mie	Topholm Bruun	Department of Clinical Immunology, Odense University Hospital, Odense, Denmark	
47	Henrik	Ullum	Statens Serum Institut, Copenhagen, Denmark	
48	Thomas	Werge	Institute of Biological Psychiatry, Mental Health Centre, Sct. Hans, Copenhagen University Hospital, Roskilde, Denmark	Department of Clinical Medicine, Faculty of Health and Medical Sciences, University of Copenhagen, Copenhagen, Denmark
49	David	Westergaard	Department of Obstetrics and Gynaecology, Copenhagen Univesity Hospital, Hvidovre Hospital, Copenhagen, Denmark	

Table S12. STROBE Statement.

	Item No	Recommendation	Page No
**Title and abstract**	1	(*a*) Indicate the study's design with a commonly used term in the title or the abstract	1
		(*b*) Provide in the abstract an informative and balanced summary of what was done and what was found	3-4
**Introduction**			
Background/rationale	2	Explain the scientific background and rationale for the investigation being reported	5-7
Objectives	3	State specific objectives, including any prespecified hypotheses	7
**Methods**			
Study design	4	Present key elements of study design early in the paper	7-8; Fig. 1
Setting	5	Describe the setting, locations, and relevant dates, including periods of recruitment, exposure, follow-up, and data collection	Table 1
Participants	6	(*a*) Give the eligibility criteria, and the sources and methods of selection of participants. Describe methods of follow-up	7-8
		(*b*) For matched studies, give matching criteria and number of exposed and unexposed	
Variables	7	Clearly define all outcomes, exposures, predictors, potential confounders, and effect modifiers. Give diagnostic criteria, if applicable	7-11
Data sources/ measurement	8*	For each variable of interest, give sources of data and details of methods of assessment (measurement). Describe comparability of assessment methods if there is more than one group	7-11, Table S3-S5
Bias	9	Describe any efforts to address potential sources of bias	7-8
Study size	10	Explain how the study size was arrived at	7-8
Quantitative variables	11	Explain how quantitative variables were handled in the analyses. If applicable, describe which groupings were chosen and why	8-10
Statistical methods	12	(*a*) Describe all statistical methods, including those used to control for confounding	11-13
		(*b*) Describe any methods used to examine subgroups and interactions	
		(*c*) Explain how missing data were addressed	8-12
		(*d*) If applicable, explain how loss to follow-up was addressed	

		(*e*) Describe any sensitivity analyses	
**Results**			
Participants	13*	(a) Report numbers of individuals at each stage of study—eg numbers potentially eligible, examined for eligibility, confirmed eligible, included in the study, completing follow-up, and analysed	7-9
		(b) Give reasons for non-participation at each stage	7-9
		(c) Consider use of a flow diagram	
Descriptive data	14*	(a) Give characteristics of study participants (eg demographic, clinical, social) and information on exposures and potential confounders	Table 1, Table S2
		(b) Indicate number of participants with missing data for each variable of interest	Table 1
		(c) Summarise follow-up time (eg, average and total amount)	Table 1
Outcome data	15*	Report numbers of outcome events or summary measures over time	Table 1
Main results	16	(*a*) Give unadjusted estimates and, if applicable, confounder-adjusted estimates and their precision (eg, 95% confidence interval). Make clear which confounders were adjusted for and why they were included	Table S6-S8
		(*b*) Report category boundaries when continuous variables were categorized	8-10; 13-21
		(*c*) If relevant, consider translating estimates of relative risk into absolute risk for a meaningful time period	
Other analyses	17	Report other analyses done—eg analyses of subgroups and interactions, and sensitivity analyses	Table S9
**Discussion**			
Key results	18	Summarise key results with reference to study objectives	21-22
Limitations	19	Discuss limitations of the study, taking into account sources of potential bias or imprecision. Discuss both direction and magnitude of any potential bias	28

Interpretation	20	Give a cautious overall interpretation of results considering objectives, limitations, multiplicity of analyses, results from similar studies, and other relevant evidence	23-27
Generalisability	21	Discuss the generalisability (external validity) of the study results	24- 28
**Other information**			
Funding	22	Give the source of funding and the role of the funders for the present study and, if applicable, for the original study on which the present article is based	31-34

*Give information separately for exposed and unexposed groups.

Note: An Explanation and Elaboration article discusses each checklist item and gives methodological background and published examples of transparent reporting. The STROBE checklist is best used in conjunction with this article (freely available on the Web sites of PLoS Medicine at http://www.plosmedicine.org/, Annals of Internal Medicine at http://www.annals.org/, and Epidemiology at http://www.epidem.com/). Information on the STROBE Initiative is available at http://www.strobe-statement.org.

References to Supplementary Material.

[1] Eggertsson HP, Jonsson H, Kristmundsdottir S, Hjartarson E, Kehr B, Masson G, et al. Graphtyper enables population-scale genotyping using pangenome graphs. Nat Genet 2017;49:1654–60. https://doi.org/10.1038/NG.3964.

[2] Kong A, Masson G, Frigge ML, Gylfason A, Zusmanovich P, Thorleifsson G, et al. Detection of sharing by descent, long-range phasing and haplotype imputation. Nat Genet 2008;40:1068–75. https://doi.org/10.1038/NG.216.

[3] Amador C, Huffman J, Trochet H, Campbell A, Porteous D, Wilson JF, et al. Recent genomic heritage in Scotland. BMC Genomics 2015;16:1–17. https://doi.org/10.1186/S12864-015-1605-2.

[4] Auton A, Abecasis GR, Altshuler DM, Durbin RM, Bentley DR, Chakravarti A, et al. A global reference for human genetic variation. Nature 2015;526:68–74. https://doi.org/10.1038/NATURE15393.

[5] Nagy R, Boutin TS, Marten J, Huffman JE, Kerr SM, Campbell A, et al. Exploration of haplotype research consortium imputation for genome-wide association studies in 20,032 Generation Scotland participants. Genome Med 2017;9:1. https://doi.org/10.1186/S13073-017-0414-4.

[6] Money D, Wilson D, Jenko J, Whalen A, Thorn S, Gorjanc G, et al. Extending long-range phasing and haplotype library imputation algorithms to large and heterogeneous datasets. Genetics Selection Evolution 2020;52:1–12. https://doi.org/10.1186/S12711-020-00558-2.

[7] Alexander DH, Novembre J, Lange K. Fast model-based estimation of ancestry in unrelated individuals. Genome Res 2009;19:1655–64. https://doi.org/10.1101/GR.094052.109.

[8] Price AL, Patterson NJ, Plenge RM, Weinblatt ME, Shadick NA, Reich D. Principal components analysis corrects for stratification in genome-wide association studies. Nat Genet 2006;38:904–9. https://doi.org/10.1038/NG1847.

[9] Gudbjartsson DF, Helgason H, Gudjonsson SA, Zink F, Oddson A, Gylfason A, et al. Large-scale whole-genome sequencing of the Icelandic population. Nat Genet 2015;47:435–44. https://doi.org/10.1038/NG.3247.

[10] Loh PR, Danecek P, Palamara PF, Fuchsberger C, Reshef YA, Finucane HK, et al. Reference-based phasing using the Haplotype Reference Consortium panel. Nat Genet 2016;48:1443–8. https://doi.org/10.1038/NG.3679.

[11] Browning SR, Browning BL. Rapid and Accurate Haplotype Phasing and Missing-Data Inference for Whole-Genome Association Studies By Use of Localized Haplotype Clustering. The American Journal of Human Genetics 2007;81:1084–97. https://doi.org/10.1086/521987.

[12] Browning BL, Zhou Y, Browning SR. A One-Penny Imputed Genome from Next-Generation Reference Panels. The American Journal of Human Genetics 2018;103:338–48. https://doi.org/10.1016/J.AJHG.2018.07.015.

[13] Mitt M, Kals M, Pärn K, Gabriel SB, Lander ES, Palotie A, et al. Improved imputation accuracy of rare and low-frequency variants using population-specific high-coverage WGS-based imputation reference panel. European Journal of Human Genetics 2017 25:7 2017;25:869–76. https://doi.org/10.1038/ejhg.2017.51.

[14] Brandlistuen RE, Kristjansson D, Alsaker E, Valen R, Birkeland E, Røyrvik EC, et al. Cohort Profile Update: The Norwegian Mother, Father and Child Cohort (MoBa). Int J Epidemiol 2025;54. https://doi.org/10.1093/IJE/DYAF139.

[15] Corfield EC, Shadrin AA, Frei O, Rahman Z, Lin A, Athanasiu L, et al. The Norwegian Mother, Father, and Child cohort study (MoBa) genotyping data resource: MoBaPsychGen pipeline v.1. BioRxiv 2024:2022.06.23.496289. https://doi.org/10.1101/2022.06.23.496289.

[16] Ge T, Chen CY, Ni Y, Feng YCA, Smoller JW. Polygenic prediction via Bayesian regression and continuous shrinkage priors. Nat Commun 2019;10. https://doi.org/10.1038/S41467-019-09718-5.

[17] Demontis D, Walters RK, Martin J, Mattheisen M, Als TD, Agerbo E, et al. Discovery of the first genome-wide significant risk loci for attention deficit/hyperactivity disorder. Nat Genet 2019;51:63–75. https://doi.org/10.1038/S41588-018-0269-7.

[18] Walters RK, Polimanti R, Johnson EC, McClintick JN, Adams MJ, Adkins AE, et al. Transancestral GWAS of alcohol dependence reveals common genetic underpinnings with psychiatric disorders. Nature Neuroscience 2018 21:12 2018;21:1656–69. https://doi.org/10.1038/s41593-018-0275-1.

[19] Purves KL, Coleman JRI, Meier SM, Rayner C, Davis KAS, Cheesman R, et al. A major role for common genetic variation in anxiety disorders. Molecular Psychiatry 2019 25:12 2019;25:3292–303. https://doi.org/10.1038/s41380-019-0559-1.

[20] Mullins N, Forstner AJ, O'Connell KS, Coombes B, Coleman JRI, Qiao Z, et al. Genome-wide association study of more than 40,000 bipolar disorder cases provides new insights into the underlying biology. Nature Genetics 2021 53:6 2021;53:817–29. https://doi.org/10.1038/s41588-021-00857-4.

[21] Jansen PR, Watanabe K, Stringer S, Skene N, Bryois J, Hammerschlag AR, et al. Genome-wide analysis of insomnia in 1,331,010 individuals identifies new risk loci and functional pathways. Nat Genet 2019;51:394–403. https://doi.org/10.1038/S41588-018-0333-3.

[22] Howard DM, Adams MJ, Clarke TK, Hafferty JD, Gibson J, Shirali M, et al. Genome-wide meta-analysis of depression identifies 102 independent variants and highlights the importance of the prefrontal brain regions. Nature Neuroscience 2019 22:3 2019;22:343–52. https://doi.org/10.1038/s41593-018-0326-7.

[23] Nagel M, Jansen PR, Stringer S, Watanabe K, De Leeuw CA, Bryois J, et al. Meta-analysis of genome-wide association studies for neuroticism in 449,484 individuals identifies novel genetic loci and pathways. Nature Genetics 2018 50:7 2018;50:920–7. https://doi.org/10.1038/s41588-018-0151-7.

[24] Nievergelt CM, Maihofer AX, Klengel T, Atkinson EG, Chen CY, Choi KW, et al. International meta-analysis of PTSD genome-wide association studies identifies sex- and ancestry-specific genetic risk loci. Nature Communications 2019 10:1 2019;10:1–16. https://doi.org/10.1038/s41467-019-12576-w.

[25] Trubetskoy V, Pardiñas AF, Qi T, Panagiotaropoulou G, Awasthi S, Bigdeli TB, et al. Mapping genomic loci implicates genes and synaptic biology in schizophrenia. Nature 2022 604:7906 2022;604:502–8. https://doi.org/10.1038/s41586-022-04434-5.

[26] Kanai M, Andrews SJ, Cordioli M, Stevens C, Neale BM, Daly M, et al. A second update on mapping the human genetic architecture of COVID-19. Nature 2023;621:E7–26. https://doi.org/10.1038/S41586-023-06355-3.

[27] Bycroft C, Freeman C, Petkova D, Band G, Elliott LT, Sharp K, et al. The UK Biobank resource with deep phenotyping and genomic data. Nature 2018 562:7726 2018;562:203–9. https://doi.org/10.1038/s41586-018-0579-z.

[28] Gagliano Taliun SA, VandeHaar P, Boughton AP, Welch RP, Taliun D, Schmidt EM, et al. Exploring and visualizing large-scale genetic associations by using PheWeb. Nat Genet 2020;52:550–2. https://doi.org/10.1038/S41588-020-0622-5.
